# Mechanistic insights into PM_2.5_-induced cardiac fibrosis involving ERS/TXNIP/NLRP3-driven pyroptosis in multiple cell types

**DOI:** 10.1093/eep/dvag015

**Published:** 2026-05-07

**Authors:** Siqi Li, Xiaohong Li, Xiaolin Han, Mengxiao Luan, Fengjiao Tan, Yumei Liu, Ruixi Zhou, Wenbo Wu, Chen Liu, Limin Zhang, Qin Wang, Yingjie Zou, Jinfeng Tan, Li Yu, Wanwei Li

**Affiliations:** School of Public Health, Shandong Second Medical University, Weifang 261053, China; School of Public Health, Shandong Second Medical University, Weifang 261053, China; “Healthy Shandong” Major Social Risk Prediction and Governance Collaborative Innovation Center, Weifang 261053, China; Key Laboratory of Health Inspection and Quarantine, Weifang 261053, China; School of Public Health, Shandong Second Medical University, Weifang 261053, China; “Healthy Shandong” Major Social Risk Prediction and Governance Collaborative Innovation Center, Weifang 261053, China; Key Laboratory of Health Inspection and Quarantine, Weifang 261053, China; School of Public Health, Shandong Second Medical University, Weifang 261053, China; “Healthy Shandong” Major Social Risk Prediction and Governance Collaborative Innovation Center, Weifang 261053, China; Key Laboratory of Health Inspection and Quarantine, Weifang 261053, China; School of Public Health, Shandong Second Medical University, Weifang 261053, China; “Healthy Shandong” Major Social Risk Prediction and Governance Collaborative Innovation Center, Weifang 261053, China; Key Laboratory of Health Inspection and Quarantine, Weifang 261053, China; School of Public Health, Shandong Second Medical University, Weifang 261053, China; “Healthy Shandong” Major Social Risk Prediction and Governance Collaborative Innovation Center, Weifang 261053, China; Key Laboratory of Health Inspection and Quarantine, Weifang 261053, China; School of Public Health, Shandong Second Medical University, Weifang 261053, China; School of Public Health, Shandong Second Medical University, Weifang 261053, China; School of Public Health, Shandong Second Medical University, Weifang 261053, China; School of Public Health, Shandong Second Medical University, Weifang 261053, China; National Institute of Environmental Health of China CDC, Beijing 100021, China; Weifang Ecological Environmental Monitoring Station, Department of Ecology and Environment of Shandong Province, Weifang 261044, China; Weifang Ecological Environmental Monitoring Station, Department of Ecology and Environment of Shandong Province, Weifang 261044, China; School of Basic Medicine, Neurologic Disorders and Regeneration Repair Lab of Shandong Higher Education, Shandong Second Medical University, Weifang 261053, China; Basic Medical College, Shandong Medical and Pharmaceutical University, Yantai 264003, China; School of Public Health, Shandong Second Medical University, Weifang 261053, China; “Healthy Shandong” Major Social Risk Prediction and Governance Collaborative Innovation Center, Weifang 261053, China; Key Laboratory of Health Inspection and Quarantine, Weifang 261053, China

**Keywords:** PM_2.5_, cardiac fibrosis, ERS, TXNIP, NLRP3, pyroptosis

## Abstract

Fine particulate matter (PM_2.5_) exposure leads to cardiovascular diseases (CVDs) by promoting cardiac fibrosis has been demonstrated. However, the mechanisms by which PM_2.5_ induces cardiac fibrosis remain unclear. Here, we confirm that the endoplasmic reticulum stress (ERS)/thioredoxin-interacting protein (TXNIP)/nucleotide-binding oligomerization domain-like receptor protein 3 (NLRP3) signaling pathway is the mechanism of action in PM_2.5_-induced cardiac fibrosis, which also plays a crucial role across multiple cell types implicated in this condition. PM_2.5_ exposure resulted in increased levels of reactive oxygen species (ROS), the occurrence of ERS, and upregulation of TXNIP expression, as well as pyroptosis and apoptosis in macrophages and cardiomyocytes, subsequently leading to the activation of MCF. The pyroptosis and apoptosis in macrophages and cardiomyocytes, along with MCF activation induced by PM_2.5_, were significantly attenuated with the inhibitors of ERS and TXNIP. We also observed that ERS and TXNIP are involved in multiple mechanisms related to oxidative stress and the inflammatory response. We provide insights into the specific mechanisms underlying PM_2.5_-induced cardiac fibrosis and suggest potential targets to control PM_2.5_-induced cardiac fibrosis.

## Graphical Abstract

**Graphical Abstract:**
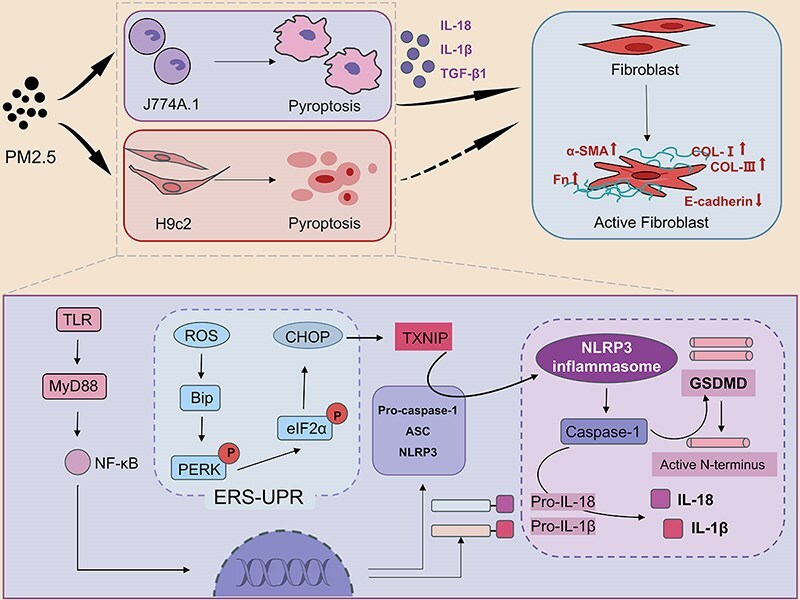

## Introduction

Cardiovascular disease (CVD) is the leading cause of global death. In 2021, CVD was responsible for ~19 million deaths worldwide [1]. In China, CVD accounted for nearly 40% of the total deaths in 2022 [2]. CVD presents a significant threat to public health and is associated with a poor prognosis; thus, it is crucial to investigate predisposing factors and underlying mechanisms of CVD. In 2019, ambient particulate matter pollution accounted for 4.5 million deaths worldwide [3]. Notably, fine particulate matter (PM_2.5_) has emerged as a major risk factor for CVD morbidity and mortality. Research indicates that for each 10 μg/m^3^ increase in PM_2.5_ exposure, CVD morbidity and mortality rise by 25.1% and 16.4%, respectively [4]. Irreversible cardiac fibrosis is one of the primary pathological causes of CVD development, leading to conditions such as coronary heart disease (CHD) and heart failure (HF) [5]. PM_2.5_ exerts its detrimental effects on the heart via pulmonary circulation and the air–blood barrier [6]. A study found that ultrafine PM particles accumulate in both the heart and lungs of mice, with concentrations in the heart surpassing those in the lungs [7]. Epidemiological studies and laboratory research suggest that PM_2.5_ exposure can induce cardiac fibrosis, resulting in cardiac dysfunction and related abnormalities [8, 9]. PM_2.5_-induced cardiac fibrosis is regulated by various cellular mechanisms and signaling pathways [10]. In addition, emerging evidence suggests that epigenetic regulation may serve as a critical link between environmental exposure and CVDs by modulating processes such as inflammation and oxidative stress [11]. However, the precise molecular mechanisms, and the role of epigenetic regulation in PM_2.5_-induced myocardial fibrosis, remain incompletely understood.

The primary characteristic of cardiac fibrosis is the abnormal proliferation of cardiac fibroblasts into myofibroblasts. Activated myofibroblasts promote excessive deposition of collagen fibers [10]. In a study examining cell lineage markers in a mouse model of myocardial infarction, it was observed that resident fibroblasts are the primary cells involved in cardiac fibrosis, which are activated into myofibroblasts. These myofibroblasts play a central role in the development of cardiac fibrosis [12]. The activation of cardiac fibroblasts is regulated by multiple mechanisms, including chemokines, cytokines, and growth factors released from the injured myocardium, as well as other various signaling pathways [13]. Bioinformatics analysis of single-cell transcriptomic data from the heart indicates that other cell types, such as cardiomyocytes, and macrophages, are closely associated with the activation of cardiac fibroblasts. These different cell types establish an intercellular communication network through growth factors, cytokines, collagen, and enzymes [14]. Therefore, investigating the specific mechanisms through which macrophages and other cell types activate cardiac fibroblasts under conditions of PM_2.5_ exposure-induced cardiac fibrosis has significant mechanistic implications for elucidating the pathophysiological pathways underlying PM_2.5_-triggered cardiac fibrosis.

The release of pro-fibrotic inflammatory mediators can be triggered by various signaling pathways, including pyroptosis, a form of programmed cell death (PCD), characterized by the rapid rupture of the cell membrane. This rupture results in the release of cellular contents and inflammatory mediators [15, 16]. Pyroptosis is a form of inflammatory cell death primarily mediated by the NOD-like receptor protein 3 (NLRP3) inflammasome, which facilitates the activation of Pro-Caspase-1 into its active form, Caspase-1. This activation subsequently triggers the maturation and release of interleukin-1β (IL-1β) and interleukin-18 (IL-18), ultimately initiating an inflammatory response [17, 18]. Researches have shown that NLRP3 inflammasome assembly plays a detrimental role in diseases such as atherosclerosis, HF, and diabetes [18, 19]. The presence of the NLRP3 inflammasome has been detected in cardiac tissue from patients with chronic CVDs and fibrosis, confirming that cardiac fibrosis may involve pyroptosis mediated by NLRP3 [20]. NLRP3 can be activated by a wide range of structurally diverse signals. For instance, upon stimulation, thioredoxin-interacting protein (TXNIP) dissociates from thioredoxin, facilitating its binding to and activation of NLRP3 [21]. In a study examining the role of TXNIP-mediated NLRP3 inflammasome activation in myocardial microvascular endothelial cells during myocardial ischemia-reperfusion injury, the knockout of TXNIP was found to reduce NLRP3 inflammasome activation and ischemia/reperfusion-induced damage. This finding demonstrates a close relationship between TXNIP and NLRP3 inflammasome activation, as well as the resulting cardiac injury [22]. However, the specific mechanism through which PM_2.5_ induces cardiac fibrosis via the TXNIP-NLRP3 pathway remains unclear and requires further investigation for confirmation.

Previous studies have confirmed the association between endoplasmic reticulum stress (ERS) and NLRP3 activation, with TXNIP playing a pivotal role in linking the two processes [23]. High levels of reactive oxygen species (ROS) and oxidative stress are key mechanisms underlying the development of CVD induced by PM_2.5_ exposure [24]. Research indicates that elevated ROS levels and oxidative stress can impair endoplasmic reticulum (ER) function, an organelle sensitive to redox changes, leading to ERS. In response to ERS, cells activate the unfolded protein response (UPR) as a stress response mechanism [25]. During the progression of the UPR, Bip is released from the transmembrane protein sensor and activates three transmembrane proteins, among which PERK is activated through phosphorylation. PERK subsequently phosphorylates the eukaryotic translation initiation factor 2 (eIF2α), further enhancing the expression of the pro-apoptotic protein C/EBP homology protein (CHOP) [26]. TXNIP acts as a downstream effector of CHOP and plays a crucial role in linking the ERS response with NLRP3 inflammasome activation. In nephrotic syndrome, the knockout of CHOP significantly inhibits the translocation of TXNIP and the activation of the NLRP3 inflammasome [27]. In the study of BDE-209-induced apoptosis in vascular endothelial cells, the ERS inhibitor 4-PBA alleviates BDE-209-induced endothelial cell apoptosis through the ERS-TXNIP-NLRP3 signaling pathway [28]. Therefore, exploring the critical role of the ERS-TXNIP-NLRP3 signaling pathway in PM_2.5_-induced cardiac fibrosis is of paramount importance.

Cardiac fibrosis represents a complex pathological process characterized by diverse cellular populations and interdependent molecular mechanisms. This study establishes *in vitro* exposure models for macrophages and cardiomyocytes to PM_2.5_, along with an indirect treatment model for cardiac fibroblasts using macrophage-conditioned medium. The critical role of the ERS-TXNIP-NLRP3 pathway is validated through the application of the ERS inhibitor 4-PBA and the TXNIP inhibitor SRI-37330. The objective is to elucidate the regulatory role, functional impact, and potential therapeutic significance of ERS and TXNIP in PM_2.5_-induced cardiac fibrosis.

## Results

### PM_2.5_ exposure causes J774A.1 cell damage, elevated ROS levels, and oxidative stress and inflammatory response

**Figure 1:**
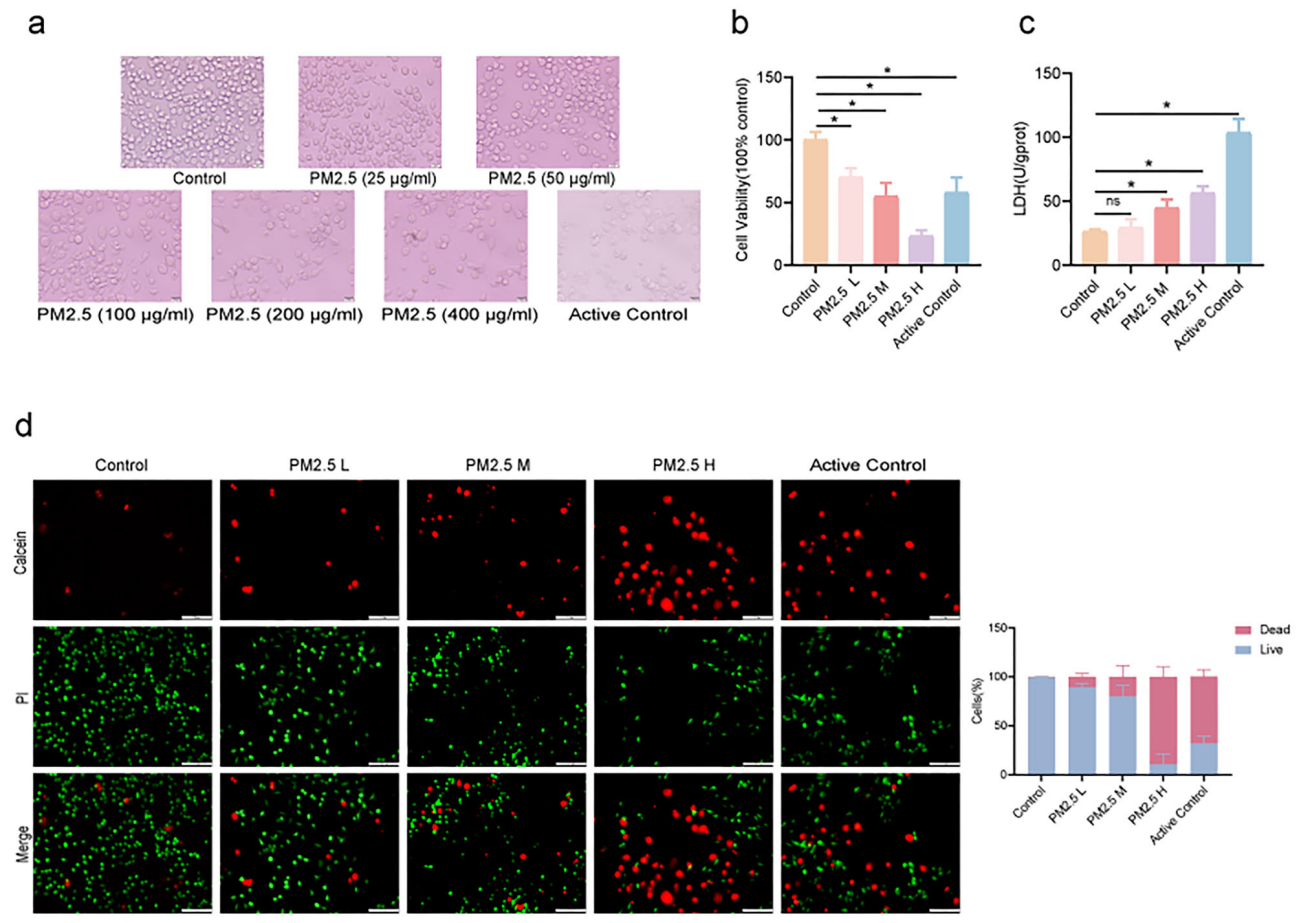
PM_2.5_ exposure causes damage to J774A.1 cells. J774A.1 cells were treated with different dose concentrations of PM_2.5_ and positive control (LPS + ATP) for 24 h. (a) Changes in cell morphology were observed in PM_2.5_ and positive control. Scale bar: 20 μm (white, lower right). (b) The cell viability changes were detected by MTT assay. (c) The LHD kit was used to detect LDH exposure in the supernatant of treated J774A.1 cells to assess cell viability in each group. (d) Calcein/PI staining assay was performed to detect the cell membrane integrity and metabolic activity of the cells to accurately assess the effect of PM_2.5_ and positive control treatments on J774A.1 cell viability. Scale bar: 100 μm (white, lower right). The data shown as mean ± SD (*n* = 3). ns, *P* ≥ .05; *P < .05.

To investigate whether PM_2.5_ exposure caused damage to J774A.1 cells, we modeled low, medium, and high PM_2.5_ exposure doses. The classical activator lipopolysaccharide (LPS) + ATP, known to induce inflammatory pyroptosis, was utilized as a positive control treatment [29]. Prior to the formal modeling of PM_2.5_ exposure and the positive control group, this study determined the PM_2.5_ treatment doses of 50, 100, and 200 μg/ml for J774A.1 cells, as well as 100 ng/ml + 3 mM for the positive control (LPS + ATP) treatment based on lactate dehydrogenase (LDH) and MTT cell viability assay (Supplementary Fig. 1). Following PM_2.5_ exposure and positive control treatment (Fig. 1a), the cell morphology of J774A.1 changed significantly. Untreated macrophages exhibited small size, full and rounded morphology, with cells closely connected. In contrast, after PM_2.5_ exposure and positive control treatment, the cells became flattened and enlarged; most cells collapsed, some ruptured and died, resulting in sparse intercellular connections. Furthermore, MTT and LDH assay results indicated a significant decrease in cell viability (*P *< .05) (Fig. 1b) and an increase in LDH released (*P *< .05) (Fig. 1c). The results of Calcein/PI staining for macrophage membrane integrity are presented in Fig. 1d. The integrity of macrophages was compromised following PM_2.5_ exposure and positive control treatment, evidenced by a decrease in the proportion of live cells (green fluorescence) and an increase in the proportion of dead cells (red fluorescence). These findings demonstrate that both PM_2.5_ exposure and positive control treatment led to significant alterations in macrophage morphology, a marked reduction in cell viability, increased LDH leakage, and overall damage to macrophages.

**Figure 2:**
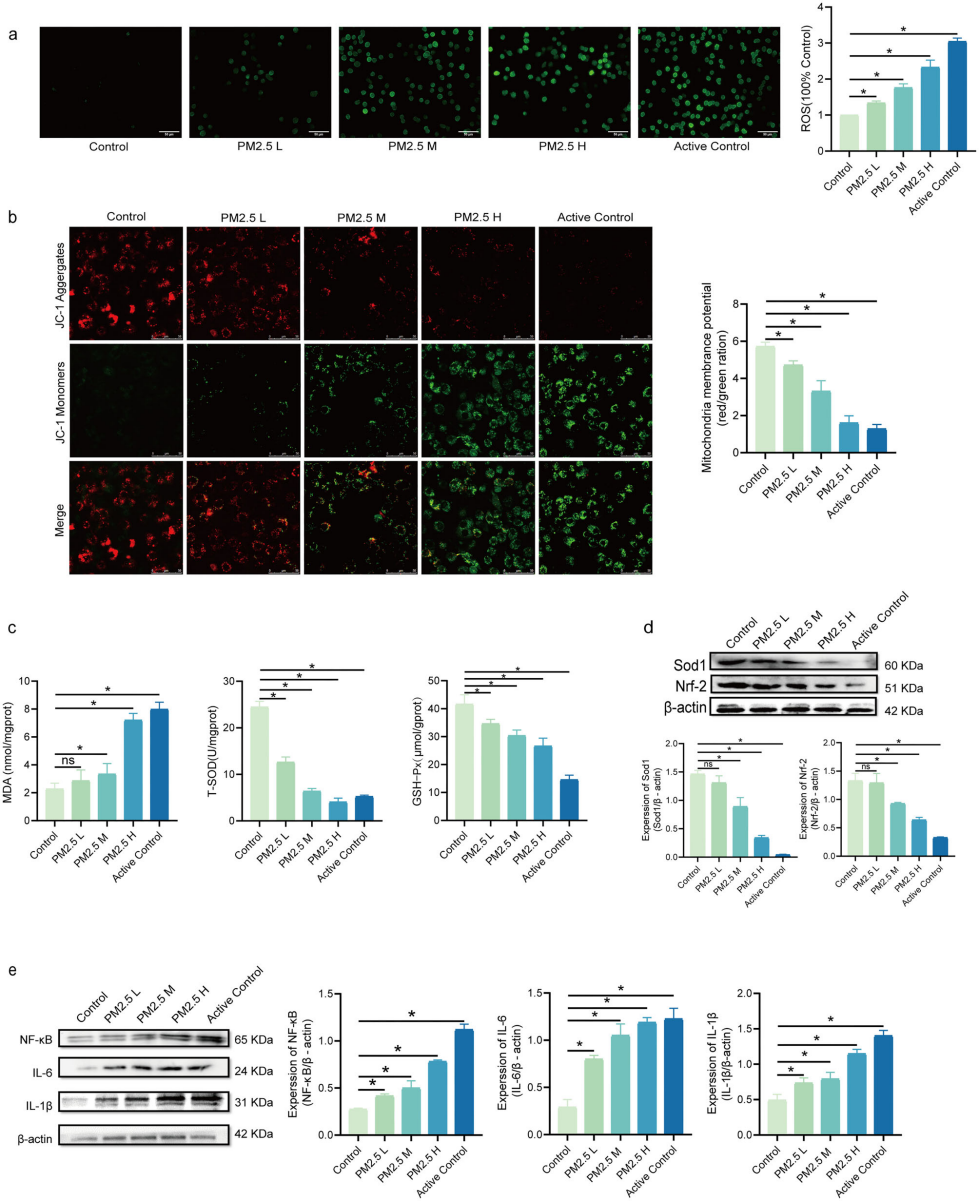
PM_2.5_ exposure causes an increase in ROS levels in J774A.1 cells, resulting in oxidative stress and inflammation. J774A.1 cells were treated with different dose concentrations of PM_2.5_ and LPS (100 ng/ml) + ATP (3 mM) for 24 h. (a) DCFH-DA fluorescent probe assay to detect ROS levels in J774A.1 cells. Scale bar: 50 μm (white, lower right). (b) JC-1 fluorescent probe detects the level of cellular mitochondrial membrane potential, reflecting the early events of ROS action on cells. Scale bar: 50 μm (white, lower right). (c) MDA, T-SOD, and GSH-Px kits were used to detect the level of oxidative stress occurring in the cells. The Western blot analysis of (d) Sod1 and Nrf-2 in the processed groups of J774A.1 cells, along with the Western blot analysis of (e) NF-κB, IL-6, and IL-1β. The data shown as mean ± SD (*n* = 3). ns, *P* ≥ .05; *P < .05.

To further investigate the potential of PM_2.5_ to induce significant levels of ROS that may lead to oxidative stress and inflammatory responses in J774A.1 macrophages, we assessed the ROS levels, oxidative stress markers, and inflammatory responses following treatment with PM_2.5_ and a positive control. Using the fluorescent probe DCFH-DA, we observed a marked increase in ROS levels in macrophages subjected to PM_2.5_ and the positive control treatment (Fig. 2a). Additionally, we employed laser confocal microscopy to evaluate alterations in the mitochondrial membrane potential of macrophages using the JC-1 probe (Fig. 2b). In the control group, JC-1 was predominantly present in its polymeric form within the mitochondria, exhibiting bright red fluorescence and minimal green fluorescence. However, following treatment with PM_2.5_ and the positive control, the mitochondrial membrane potential was diminished, preventing JC-1 from maintaining its polymeric form in the mitochondrial matrix. Consequently, the intensity of red fluorescence in the mitochondria significantly decreased, while green fluorescence in the cytoplasm markedly increased. Moreover, our findings indicated that exposure to PM_2.5_ and the positive control treatment led to elevated MDA levels and a significant reduction in GSH-Px and T-SOD levels in J774A.1 (Fig. 2c). Western blot analysis (Fig. 2d) revealed increased expression of Sod1 and Nrf-2 protein in macrophages. These experimental results collectively suggest that J774A.1 macrophages experience oxidative stress. Furthermore, we evaluated the expression levels of typical inflammatory proteins, including NF-κB, IL-6, and IL-1β, in macrophages. The results from Western blot analysis, illustrated in Fig. 2e, demonstrated that these inflammatory markers were elevated following treatment with PM_2.5_ and the positive control. Collectively, these findings indicate that PM_2.5_ exposure contributes to increased ROS levels in macrophages, thereby promoting oxidative stress and inflammatory responses.

### PM_2.5_ exposure causes J774A.1 to undergo ERS and leads to pyroptosis and apoptosis

**Figure 3:**
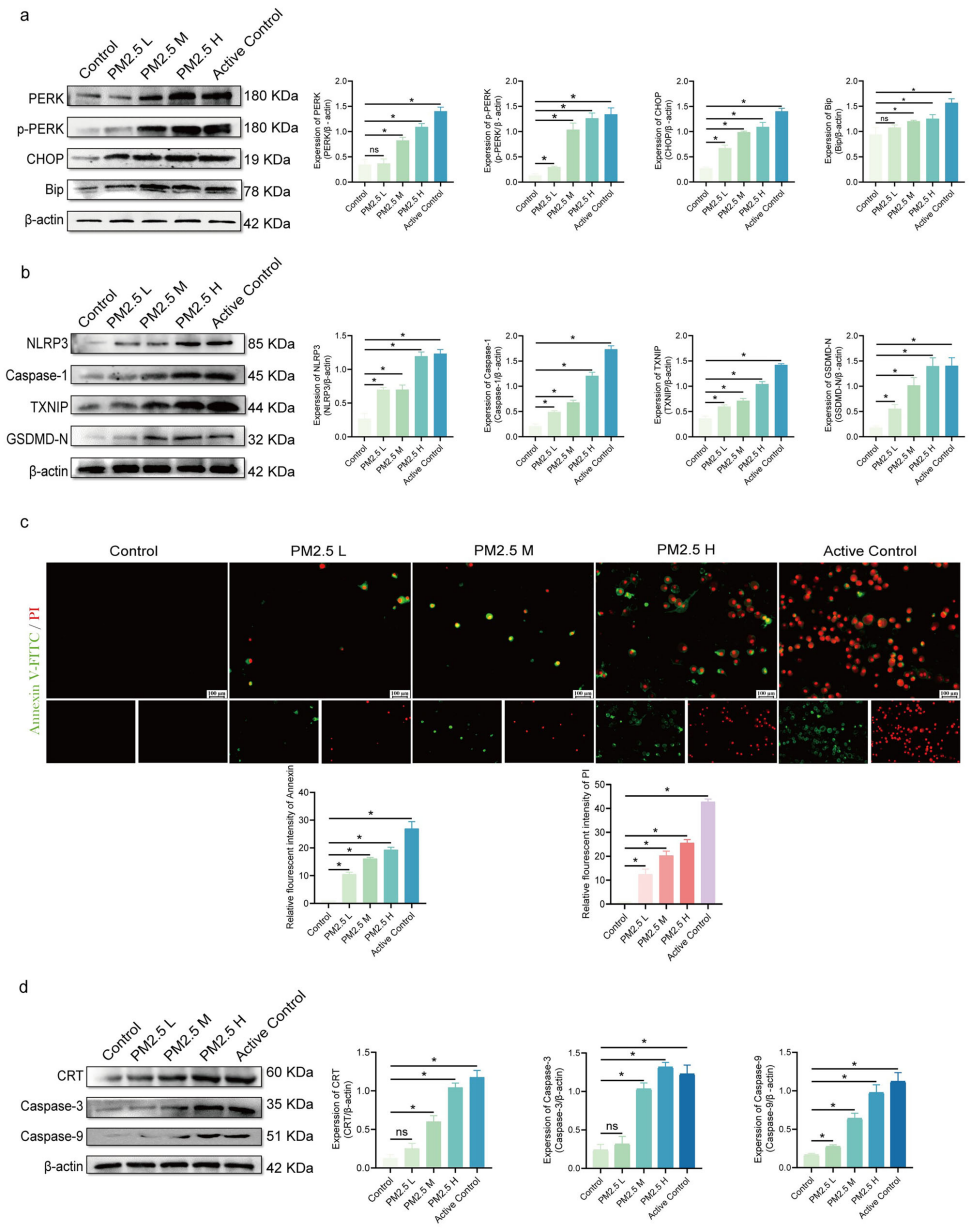
PM_2.5_ exposure causes J774A.1 cell ERS activation, activates TXNIP, and leads to pyroptosis and apoptosis. J774A.1 cells were treated with different concentrations of PM_2.5_ and LPS (100 ng/ml) + ATP (3 mM) for 24 h. (a) Western blot analysis of the expression levels of PERK, p-PERK, CHOP, and Bip proteins in the ERS response in J774A.1 cells and (b) the expression levels of TXNIP protein and pyroptotic markers NLRP3, Caspase-1, and GSDMD-N proteins. (c) The Annexin V/PI staining method for detecting the level of cell apoptosis. Scale bar: 100 μm (white, lower right). (d) Western blot analysis of CRT, Caspase-3, and Caspase-9 in each group of cells. The data shown as mean ± SD (*n* = 3). ns, *P* ≥ .05; *P < .05.

ERS can be activated by elevated levels of ROS [30, 31]. To investigate whether PM_2.5_ exposure induces ERS in J774A.1 cells, we detected ERS levels by western blotting (WB) detection of changes in the protein expression levels of PERK, p-PERK, CHOP, and Bip. The results showed that J774A.1 cells were exposed to PM_2.5_ and treated with LPS + ATP, which elevated the expression of Bip and caused the activation of PERK, a transmembrane protein, resulting in an increase in the expression level of p-PERK, which ultimately enhanced the expression of CHOP proteins (Fig. 3a). These results indicate that PM_2.5_ exposure caused ERS in J774A.1 cells. Notably, TXNIP protein expression was elevated (Fig. 3b), and as a possible connection between ERS and scorch death leading to scorch death occurs. The results showed that the expression of pyroptosis-related proteins NLRP3, Caspase-1, and GSDMD-N was increased (Fig. 3b). The results of Annexin V/PI co-staining fluorescence staining are shown in Fig. 3c. After treatment with PM_2.5_ exposure and positive control, the red and green fluorescence were significantly enhanced, and J774A.1 cells underwent apoptosis. In addition, CRT, Caspase-3, and Caspase-9 protein expression was significantly elevated (Fig. 3d). The results indicate that after exposure to PM_2.5_, J774A.1 cells undergo ERS, which activates the TXNIP-NLRP3 pathway, leading to the induction of pyroptosis and apoptosis.

### PM_2.5_ induces the release of inflammatory and pro-fibrotic factors from J774A.1 and causes myocardial fibroblast activation

**Figure 4:**
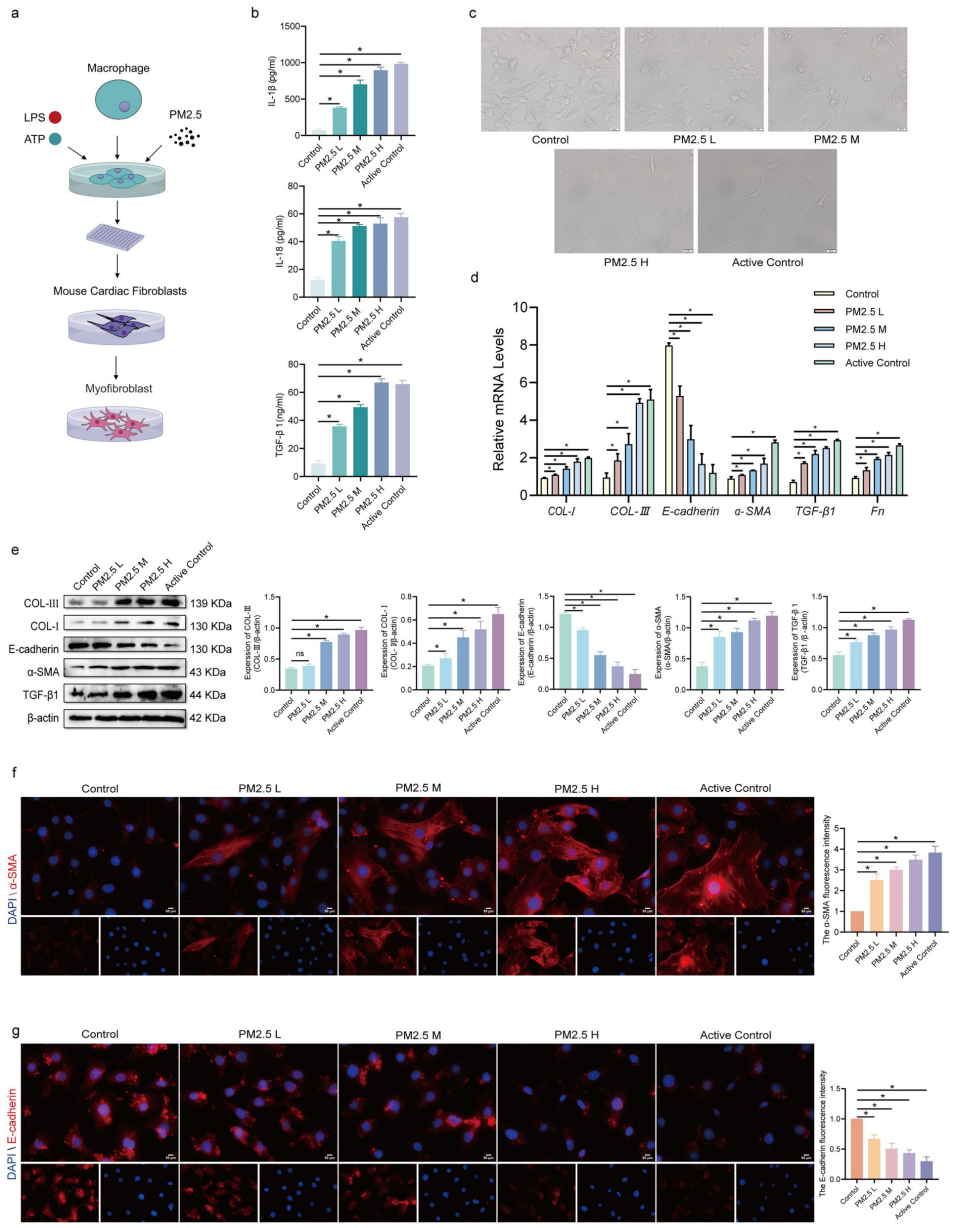
PM_2.5_ exposure causes cytokine release from J774A.1 cells to induce MCF cell activation. The supernatants from macrophages treated with varying doses of PM_2.5_ in combination with LPS (100 ng/mL) and ATP (3 mM) were collected, and cytokine levels were analyzed. These supernatants were subsequently used for indirect co-treatment experiments with cardiac fibroblasts. (a) Flowchart for establishing an indirect co-culture model of macrophages and cardiac fibroblasts. (b) The levels of IL-1β, IL-18, and TGF-β1 in the supernatant of macrophages. After treatment with the collected macrophage supernatant for 24 h, investigate (c) changes in the morphology of cardiac fibroblasts (CFs) (scale bar: 20 μm) and the (d) mRNA expression levels of COL-III, COL-I, E-cadherin, α-SMA, TGF-β1, and FN. (e) The expression levels of COL-III, COL-I, E-cadherin, α-SMA, and TGF-β1 proteins. Changes in (f) α-SMA and (g) E-cadherin fluorescence intensity. Scale bar: 50 μm (white, lower right). The data shown as mean ± SD (*n* = 3). ns, *P* ≥ .05; *P < .05.

Release of IL-18 and IL-1β inflammatory factors is an important marker for the onset of pyroptosis and can lead to multi-tissue and multi-organ damage [32]. In addition, various cytokines, including inflammatory factors and pro-fibrotic factors, play a critical role in the crosstalk between cells [33]. To investigate whether J774A.1 cells treated with PM_2.5_ exposure and LPS + ATP release relevant cytokines, we collected the supernatant of J774A.1-treated cells and completed the ELISA assay (Fig. 4a). The results of the ELISA assay for IL-18 and IL-1β inflammatory factors and TGF-β1 fibrosis-promoting factor in the macrophage supernatant are shown in Fig. 4b. The levels of these factors were significantly elevated in the PM_2.5_ group and the positive control group (*P *< .05).

To further investigate the effect of cytokines released by macrophages induced by PM_2.5_ on the activation of cardiac fibroblasts, we performed relevant assays on cardiac fibroblasts cultured with macrophage-conditioned medium (Fig. 4a). After the MTT assay, we determined that the macrophage supernatant treatment time for cardiac fibroblasts was 24 h (Supplementary Fig. 2). As shown in Fig. 4c, the control group MCF cells exhibited a monolayer of spindle-shaped cells. After exposure to PM_2.5_ and treatment with the supernatant from the positive control group, the spindle-shaped MCF cells transformed into myofibroblasts, characterized by a large surface area and various protrusions on the plasma membrane. In addition, the mRNA expression levels and protein expression of COL-I, COL-III, α-SMA, and TGF-β1 were significantly elevated, while the mRNA levels of FN were increased. Conversely, the mRNA levels and protein expression of E-cadherin were significantly reduced (Fig. 4d and e). The immunofluorescence results for α-SMA and E-cadherin in MCF cells, as shown in Fig. 4f and g, demonstrate that following exposure to PM_2.5_ and treatment with macrophage supernatant from the positive control group, the fluorescence intensity of α-SMA was enhanced, whereas the fluorescence intensity of E-cadherin was reduced. The results above indicate that PM_2.5_ exposure leads to macrophages releasing inflammatory and pro-fibrotic factors, thereby inducing the activation of MCFs into myofibroblasts.

### PM_2.5_ exposure leads to elevated ROS levels, oxidative stress, and inflammation in H9c2 cells

**Figure 5:**
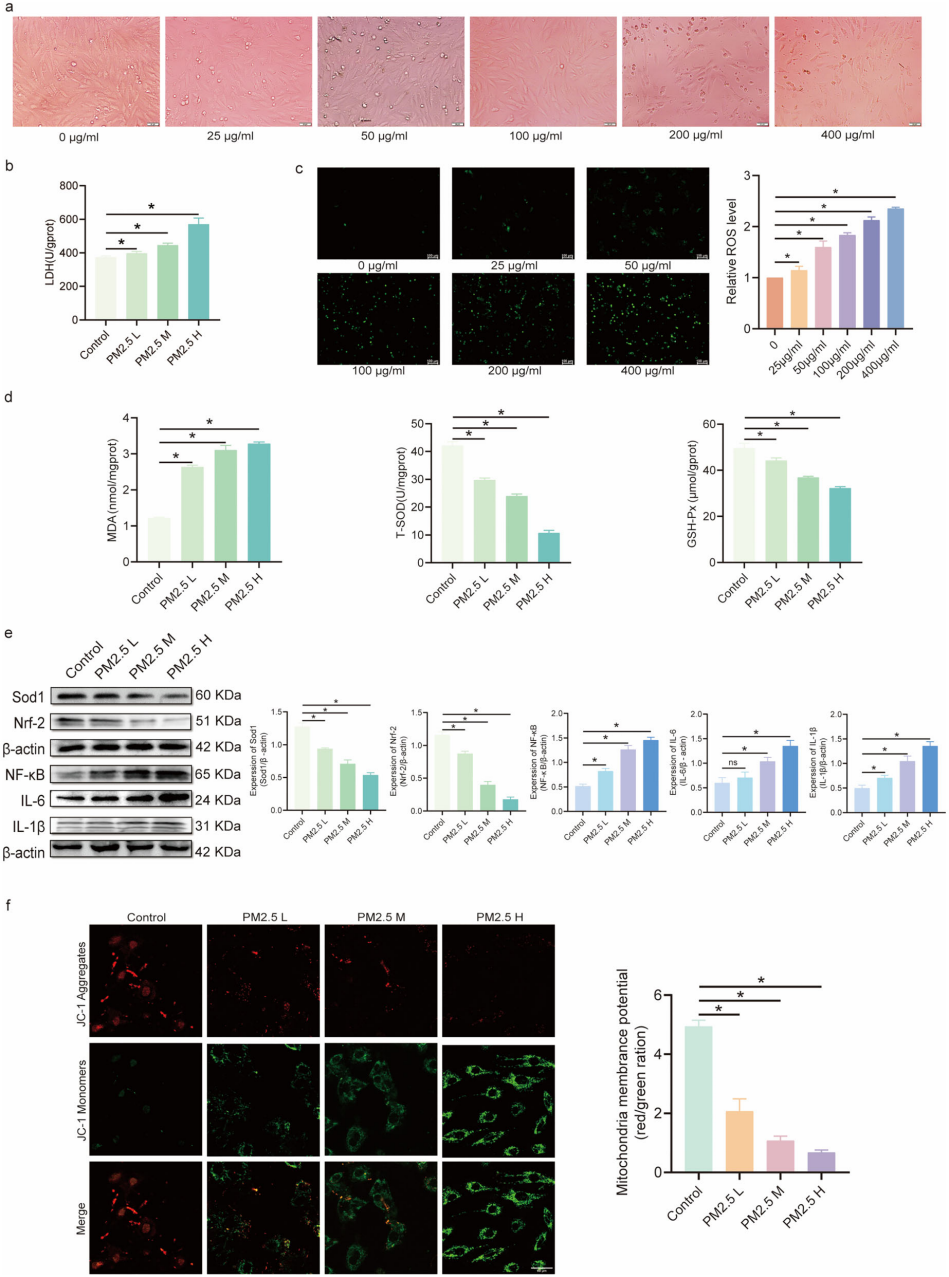
PM_2.5_ exposure causes damage to H9c2 cells. The H9c2 cells were treated with different concentrations of PM_2.5_ for 24 h, and the extent of cardiomyocyte damage was assessed. (a) The morphological changes of H9c2 cells after treatment with different concentrations of PM_2.5_ and the control group. Scale bar: 50 μm (white, lower right). (b) The changes in LDH release levels in H9c2 cells following exposure to PM_2.5_. (c) The effect of different doses of PM_2.5_ exposure on cellular ROS levels. Scale bar: 100 μm (white, lower right). (d) The changes in the levels of MDA, T-SOD, and GSH-Px in each group of H9c2 cells after exposure to PM_2.5_. (e) The changes in the expression levels of Sod1, Nrf-2, NF-κB, IL-6, and IL-1β proteins in H9c2 cells after exposure to different doses of PM_2.5_ compared to the control group. (f) The change in mitochondrial membrane potential of H9c2 cells induced by PM_2.5_. Scale bar: 50 μm (white, lower right). The data shown as mean ± SD (*n* = 3). ns, *P* ≥ .05; *P < .05.

Cardiomyocytes are one of the most abundant cell types in cardiac tissue, and their damage plays a critical role in cardiac fibrosis [34]. Therefore, it is essential to investigate whether PM_2.5_ can cause damage to cardiomyocytes. This study investigates whether exposure to PM_2.5_ induces damage to cardiomyocytes by examining morphological changes, alterations in cell viability, and variations in LDH levels in H9c2 cells. The H9c2 cells in the control group exhibit tight cell–cell junctions and grow in a spindle-shaped morphology, resembling elongated myocytes that adhere to the substrate (Fig. 5a). Following PM_2.5_ exposure, slight deposition of particulate matter was observed. Exposure to different doses of PM_2.5_ resulted in significant morphological changes in cardiomyocytes. Cell growth became sparse, with some cell membranes exhibiting rupture. Additionally, round, bright, dead cardiomyocytes were observed in the field of view. After exposure to PM_2.5_, the levels of LDH in cells were elevated (Fig. 5b), and cell viability was reduced (Supplementary Fig. 3). These results indicate that PM_2.5_ exposure causes severe damage to cardiomyocytes.

We further investigated the effects of PM_2.5_ exposure on ROS levels, oxidative stress, and inflammatory responses in H9c2 cells. The results of ROS fluorescence intensity in H9c2 cells (Fig. 5c) showed that PM_2.5_ exposure leads to an increase in ROS levels. The activity of T-SOD, MDA content, and GSH-Px levels are shown in Fig. 5d. After exposure to PM_2.5_, the H9c2 cells MDA content increased, while the levels of T-SOD and GSH-Px decreased. The results of the expression levels of oxidative stress and inflammation-related proteins (Fig. 5e) indicate a decrease in the expression of Nrf-2 and Sod1, while the expression levels of NF-κB, IL-6, and IL-1β are elevated. The results of mitochondrial membrane potential (JC-1) fluorescence intensity (Fig. 5f) indicate a significant decrease in mitochondrial membrane potential following exposure to PM_2.5_. The results indicate that exposure to PM_2.5_ leads to myocardial cell damage, increased ROS levels, and the occurrence of oxidative stress and inflammatory responses.

### PM_2.5_ causes ERS activation in H9c2 cells and induces pyroptosis and apoptosis

**Figure 6:**
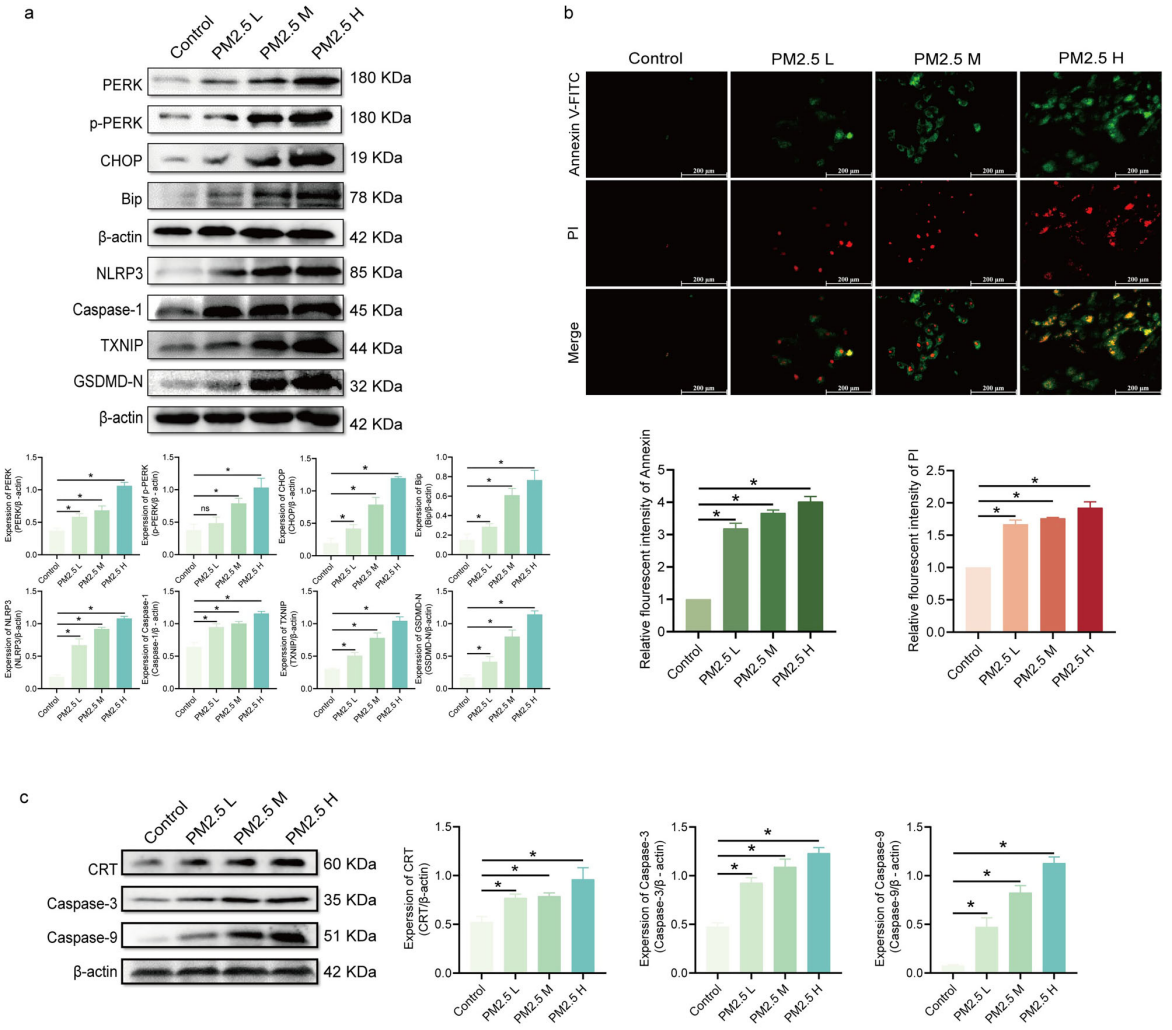
PM_2.5_ exposure causes H9c2 cells ERS activates, increases the expression level of TXNIP, and leads to pyroptosis and apoptosis. Treatment of H9c2 cells with different doses of PM_2.5_ for 24 h. (a) The expression levels of ER stress-related proteins (PERK, p-PERK, CHOP, and Bip), TXNIP, and pyroptosis-related proteins (NLRP3, Caspase-1, and GSDMD-N) in cardiomyocytes were assessed using Western blotting. (b) The Annexin V/PI double staining method was used to detect changes in the level of apoptosis in cardiomyocytes. Scale bar: 200 μm (white, lower right). Additionally, panel (c) assessed the changes in the protein expression levels of CRT, Caspase-3, and Caspase-9 in cardiomyocytes. The data shown as mean ± SD (*n* = 3). ns, *P* ≥ .05; *P < .05.

To further investigate the potential involvement of the ERS/TXNIP/NLRP3 signaling pathway in PM_2.5_-induced myocardial cell damage, this study will utilize methods such as Western blotting to assess the changes in relevant indicators. The results showed that after exposure to PM_2.5_, the expression levels of ERS-related proteins in cardiomyocytes, including PERK, p-PERK, CHOP, and Bip, were elevated. Additionally, the expression of TXNIP protein was increased, along with elevated levels of pyroptosis-related proteins NLRP3, Caspase-1, and GSDMD-N (Fig. 6a). The results of Annexin V/PI co-staining fluorescence are shown in Fig. 6b. After exposure to PM_2.5_, both red and green fluorescence significantly increased, indicating apoptosis of the cardiomyocytes. Additionally, the expression levels of CRT, Caspase-3, and Caspase-9 proteins were significantly increased (Fig. 6c). The results indicate that after exposure to PM_2.5_, myocardial cells undergo ERS and activate the TXNIP-NLRP3 pathway, thereby inducing pyroptosis and apoptosis.

### ERS and TXNIP inhibitors inhibit pyroptosis in J774A.1 cells and H9c2 cells, and the activation of MCF cells

**Figure 7:**
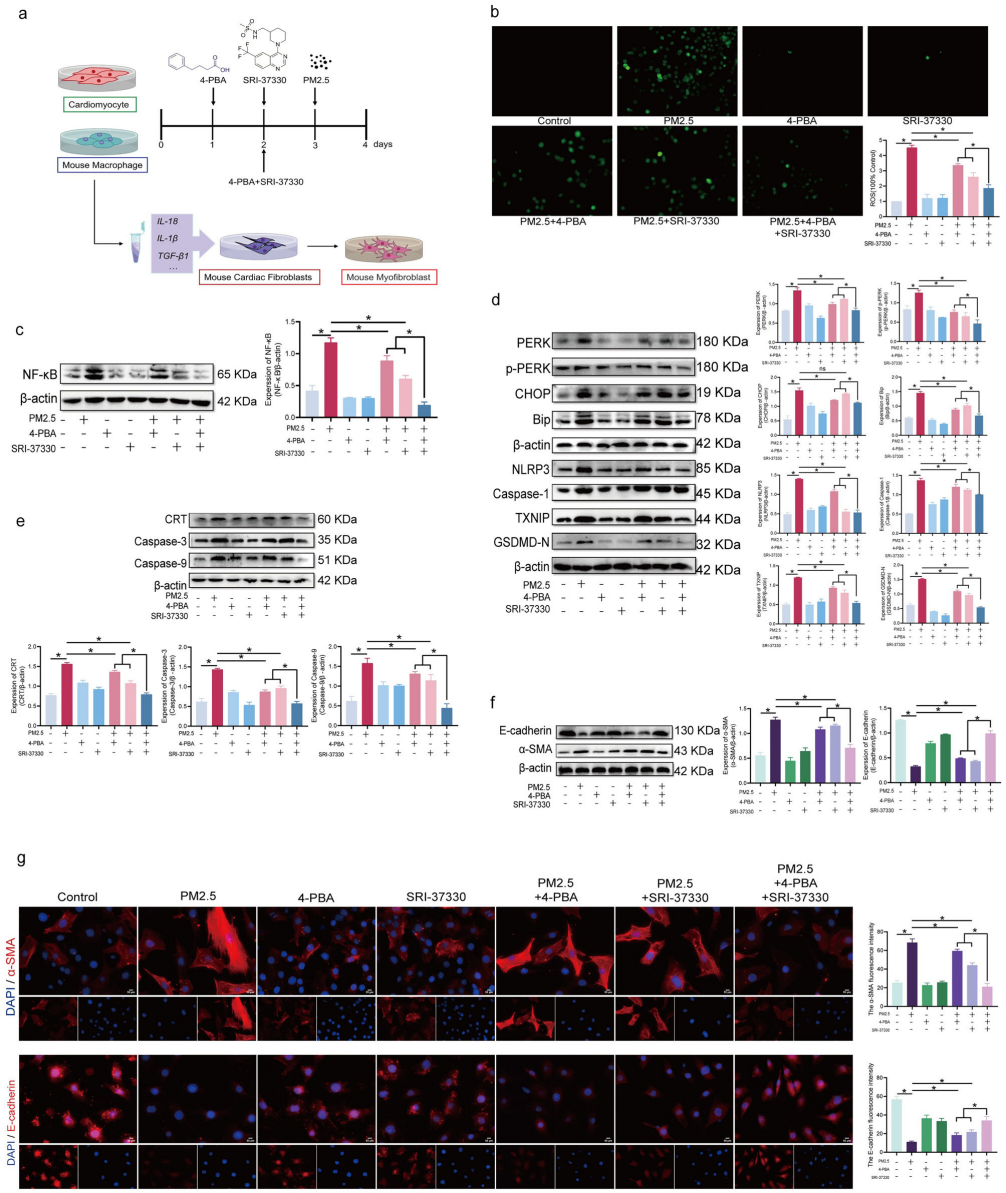
ERS and TXNIP play a key role in pyroptosis and apoptosis in J774A.1 cells induced by PM_2.5_ exposure, and also contribute to J774A.1-MCF crosstalk. Add the classic inhibitors of ERS and TXNIP, 4-PBA and SRI-37330, and treat macrophages for 24 h. Assess macrophage injury and changes in signaling pathways (a–e). (a) A time-flow diagram for the treatment of macrophages, cardiomyocytes, and the establishment of an indirect co-treatment model of macrophages and cardiac fibroblasts with PM_2.5_, 4-PBA, and SRI-37330. (b) The changes in ROS levels in macrophages after treatment. Scale bar: 50 μm (white, lower right). (c) The changes in the protein expression levels of NF-κB. (d) The expression levels of ERS-related proteins (PERK, p-PERK, CHOP, and Bip), TXNIP, and pyroptosis-related proteins (NLRP3, Caspase-1, and GSDMD-N) were analyzed for changes. (e) The changes in the expression levels of CRT, Caspase-3, and Caspase-9 proteins. The culture supernatant of macrophages exposed to PM_2.5_, treated with 4-PBA and SRI-37330, was collected and used to treat cardiac fibroblasts for 24 h. The role of ERS and TXNIP in the activation of cardiac fibroblasts induced by these treatments was then assessed (f and g). The processed results of E-cadherin and α-SMA in cardiac fibroblasts, as detected by (f) Western blotting and (g) immunofluorescence analysis. Scale bar: 50 μm (white, lower right). The data shown as mean ± SD (*n* = 3). ns, *P* ≥ .05; *P < .05.

To further investigate whether ERS and TXNIP play key roles in PM_2.5_-induced cardiac fibrosis, this study employed the classical ERS inhibitor 4-PBA and the TXNIP inhibitor SRI-37330 [35, 36]. A flowchart of the treatment of J774A.1, H9c2, and MCF with PM_2.5_ and the inhibitor is shown in Fig. 7a. This study determined the treatment dose of J774A.1 for 4-PBA and SRI-37330 by the MTT cell viability assay (Supplementary Fig. 4). After treatment with 4-PBA and SRI-37330, the ROS levels in macrophages were significantly reduced compared to the PM_2.5_ exposure group (Fig. 7b), and the expression level of NF-κB protein is reduced (Fig. 7c). In addition, we examined the expression of the ERS/TXNIP/NLRP3 signaling pathway, which is the focus of this study, within macrophages. As shown in Fig. 7d, the expression levels of macrophage ERS proteins PERK, p-PERK, CHOP, and Bip were reduced, along with a decrease in the expression of the mediator protein TXNIP. Additionally, the expression levels of pyroptosis-related proteins NLRP3, Caspase-1, and GSDMD-N were also reduced. The expression of apoptosis-related proteins CRT, Caspase-3, and Caspase-9 in macrophages is reduced (Fig. 7e).

Our research has confirmed the existence of signal crosstalk between macrophages and MCF, but it remains to be further determined whether ERS and TXNIP play a role in this crosstalk. We collected the supernatants from macrophages in each group and indirectly treated MCF, followed by the detection of fibrosis markers in MCF. The time of supernatant treatment of MCF cells was determined to be 24 h after the MTT assay (Supplementary Fig. 5). The results showed that, compared to the MCF and PM_2.5_ groups treated with 4-PBA and SRI-37330, the expression of α-SMA protein was reduced, while the expression of E-cadherin protein was increased (Fig. 7f). The immunofluorescence results of α-SMA and E-cadherin (Fig. 7g) demonstrated a decrease in α-SMA fluorescence intensity and an increase in E-cadherin fluorescence intensity. The fluorescence intensity of CTGF was significantly reduced after treatment with 4-PBA and SRI-37330 (Supplementary Fig. 6). Additionally, the levels of IL-18, IL-1β, and TGF-β1 in the macrophage supernatant, as measured by ELISA, were significantly reduced after treatment with the inhibitor (Supplementary Fig. 7). The above results indicate that ERS and TXNIP play key roles in the signaling pathways involved in PM_2.5_-induced macrophage-cardiac fibroblast activation, and they represent crucial targets in PM_2.5_-induced cardiac fibrosis and intercellular crosstalk.

**Figure 8:**
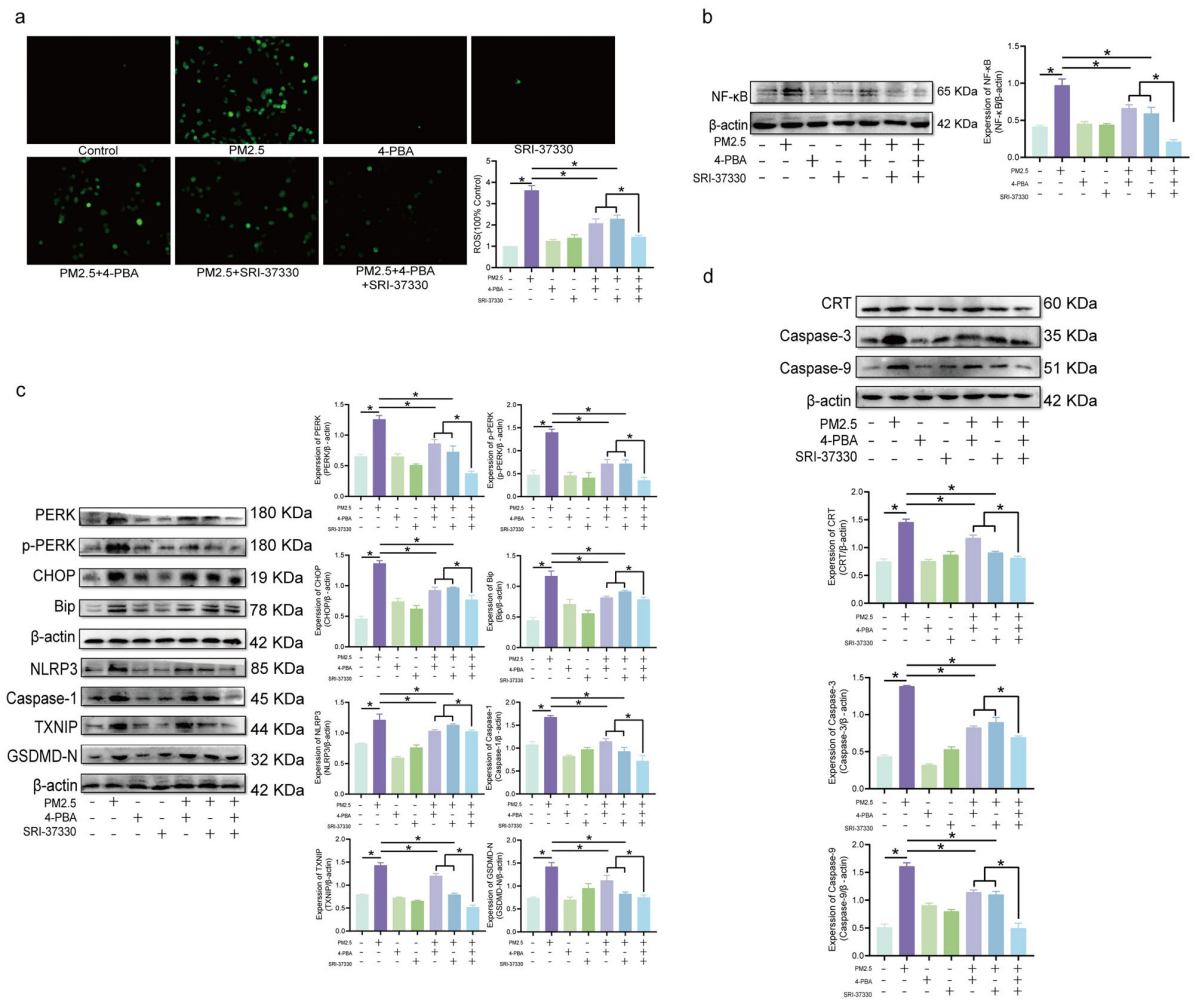
ERS and TXNIP play a key role in pyroptosis and apoptosis in H9c2 cells caused by PM_2.5_ exposure. The classic inhibitors of ERS and TXNIP, 4-PBA and SRI-37330, were added to H9c2 cardiomyocytes, which were treated for 24 h. The following parameters were assessed: (a) changes in ROS levels across different groups (scale bar: 50 μm), (b) alterations in NF-κB protein expression, and (c) variations in the expression levels of ERS-related proteins (PERK, p-PERK, CHOP, and Bip), TXNIP, and pyroptosis-associated proteins (NLRP3, Caspase-1, and GSDMD-N). And (d) the changes in the expression levels of CRT, Caspase-3, and Caspase-9 proteins. The data shown as mean ± SD (*n* = 3). ns, *P* ≥ .05; *P < .05.

The cardiomyocytes are also an important part of our research. After the MTT cell viability assay (Supplementary Fig. 8), we applied 4-PBA and SRI-37330 treatments to the cardiomyocytes and performed related assays. 4-PBA and SRI-37330 significantly attenuate the increase in ROS levels (Fig. 8a) and NF-κB protein expression (Fig. 8b) in H9c2 cells following PM_2.5_ exposure. It is noteworthy that the expression levels of the ERS proteins PERK, p-PERK, CHOP, and Bip, as well as the expression of the TXNIP protein, were decreased in H9c2 cells. Additionally, the expression levels of pyroptosis-related proteins NLRP3, Caspase-1, and GSDMD-N were also reduced (Fig. 8c). Additionally, 4-PBA and SRI-37330 can significantly reduce the increased expression of CRT, Caspase-3, and Caspase-9 proteins in H9c2 cells following exposure to PM_2.5_ (Fig. 8d). The above results indicate that ERS and TXNIP inhibitors can significantly mitigate the excessive ROS expression, inflammatory response, ERS, and TXNIP-NLRP3-mediated pyroptosis and apoptosis in H9c2 cells induced by PM_2.5_ exposure.

## Discussion

Given the established relationship between PM_2.5_ and CVD, along with the critical role of cardiac fibrosis in this context, this study develops an *in vitro* model of PM_2.5_ exposure to elucidate the mechanisms underlying of PM_2.5_-induced cardiac fibrosis and the significant involvement of the ERS/TXNIP/NLRP3 pathway across various cell types associated with cardiac fibrosis. As anticipated, investigating the injury mechanisms related to PM_2.5_ exposure is crucial for mitigating its detrimental effects on the lungs [37, 38], heart [39, 40], brain [41], and other organs. Previous research has predominantly focused on oxidative stress, inflammatory responses, and apoptosis as mechanisms of PM_2.5_-induced damage, particularly concerning cardiac fibrosis [42]. Recent studies have suggested that programmed necrosis, particularly that mediated by NLRP3, contributes to multi-organ injury. In this study, we examine the pivotal role of the ERS/TXNIP/NLRP3 signaling pathway in PM_2.5_-induced cardiac fibrosis and its significant involvement in cell-to-cell action, an area that remains underexplored. Our findings provide compelling evidence for potential therapeutic or preventive strategies against PM_2.5_-induced cardiac fibrosis and associated CVD.

Firstly, we established a macrophage model of PM_2.5_ exposure and treated it with LPS and ATP, classic pyroptosis activators, as the positive control group. Preliminary observations on the effects of PM_2.5_ on J774A.1 cells damage indicate that exposure to PM_2.5_ induces significant morphological changes in J774A.1 cells, reduces cell viability, increases LDH leakage, and causes cell membrane rupture and cell death. A fundamental insight is that high levels of ROS serve as the initial mechanism underlying damage induced by PM_2.5_. The excessive generation of ROS can further trigger oxidative stress and inflammatory responses [43–45]. This study further investigates this and the results indicate that exposure to PM_2.5_ leads to an increase in ROS levels in J774A.1 cells, causing an imbalance between oxidation and antioxidation, triggering oxidative stress and inflammatory responses. These findings are generally consistent with related research results [46]. In addition to directly damaging cellular proteins, lipids, and DNA—thereby impairing the function of redox-sensitive organelles such as the ER—ROS have also been shown to induce epigenetic modifications, including histone acetylation and aberrant DNA methylation [31, 47]. These changes may contribute to the sustained transcription of stress-responsive genes [48]. Accordingly, the elevated ROS levels observed in this study may not only trigger ER stress but also potentially prolong its activation through epigenetic regulation.

The ER is a critical organelle for maintaining normal cellular function in multicellular organisms. Disturbances in its homeostasis and the occurrence of ERS are associated with myocardial ischemia, cardiac hypertrophy, HF, and potentially atherosclerosis [49]. The ER requires a highly oxidative environment to facilitate disulfide bond formation, which is essential for proper protein synthesis, folding, and trafficking. However, when the levels of ROS exceed the ER’s buffering capacity, the formation of disulfide bonds is disrupted by the disturbance of the redox homeostasis within the ER lumen. This disruption leads to the accumulation of misfolded or unfolded proteins in the ER, resulting in ERS [50]. To counteract ERS, cells trigger the UPR, which promotes the refolding of proteins and activates ER-associated degradation (ERAD) to eliminate irreparable proteins, thereby restoring ER homeostasis. During this process, the calcium-dependent molecular chaperone Bip (glucose-regulated protein 78, also known as GRP78) within the ER dissociates from three transmembrane proteins, including PERK, and subsequently activates them. Upon activation, p-PERK further induces the phosphorylation of eIF2α, which in turn leads to an increase in the expression of CHOP protein through the activation of ATF4 [25]. Previous studies have suggested that TXNIP may play a role in linking ERS and the NLRP3 inflammasome, and that it can be activated by the PERK signaling pathway during ERS [23]. In this study, the expression levels of macrophage PERK, p-PERK, Bip, and CHOP proteins were elevated after exposure to PM_2.5_, and these levels were reduced following the application of the ERS inhibitor 4-PBA. It is noteworthy that while 4-PBA inhibits the expression of ERS in macrophages, the protein expression level of TXNIP is also reduced. These data suggest that PM_2.5_ exposure induces ERS in macrophages and modulates the expression of TXNIP through ERS regulation.

TXNIP has been identified as a binding partner of TRX in yeast two-hybrid screening. It interacts with TRX through the α-arresting domain and inhibits its activity, thereby regulating cellular redox signaling [23]. Currently, increasing attention is being paid to the role of TXNIP in activating the NLRP3 inflammasome, thereby triggering inflammatory signaling in various diseases [22]. The activation of TXNIP-NLRP3 has been shown to play a critical role in cardiac fibrosis and associated CVDs [22, 51]. The mechanism underlying its role in PM_2.5_-induced cardiac fibrosis has not yet been fully elucidated. In this study, PM_2.5_ exposure was consistent with the LPS + ATP positive control group, resulting in an elevated expression of macrophage NLRP3, Caspase-1, and GSDMD-N proteins. The treatment with SRI-37330 significantly reduced the activation of the NLRP3 inflammasome in macrophages exposed to PM_2.5_. Our results indicate that TXNIP activation is closely associated with NLRP3, and plays a significant role in macrophage injury induced by PM_2.5_ exposure.

We further explored the role of macrophage activation in the activation of myofibroblasts following exposure to PM_2.5_. Previous studies have shown that myofibroblasts are the primary effector cells in cardiac fibrosis. Macrophages, as a source of inflammatory and fibrotic mediators, can produce and secrete a large number of pro-inflammatory cytokines and pro-fibrotic growth factors, thereby indirectly activating fibrotic pathways [52]. However, there is a research gap regarding the role of J774A.1 cells and the ERS/TXNIP/NLRP3 signaling pathway in PM_2.5_-induced cardiac fibrosis. To address this gap, we conducted a study using an indirect co-culture model of J774A.1 cells and MCF cells. The research results indicate that the levels of IL-18, IL-1β, and TGF-β1 in the J774A.1 cell supernatants treated with PM_2.5_ and the positive control group were elevated, leading to the release of a significant amount of inflammatory and pro-fibrotic factors. The release of these factors is considered one of the key elements in the transformation of MCF cells into myofibroblasts [53]. TGF-β exerts widespread direct effects on fibroblasts. As a growth factor with significant fibroblast-activating properties, it promotes the transcription of α-SMA in fibroblasts, inhibits the expression of E-cadherin, and enhances the synthesis of ECM proteins in activated fibroblasts [54]. In addition, ECM proteins in fibrotic hearts play a crucial role in regulating cellular responses and mediating the signaling pathways involved in fibrosis. Notably, the secretion of major fibrillar collagens, including COL-I and COL-III, serves as a hallmark of cardiac fibrosis [55]. In this study, indirect co-treatment of J774A.1 cells after PM_2.5_ exposure results in the elevated expression of α-SMA, COL-I, COL-III, and TGF-β1 in MCF cells, while decreasing the expression of E-cadherin. This process promotes the transformation of MCF cells into myofibroblasts, thereby activating fibrosis. It is noteworthy that treatment with 4-PBA and SRI-37330 significantly reduced the protein level and fluorescence intensity of α-SMA, while increasing the protein level and fluorescence intensity of E-cadherin, effectively alleviating the fibrotic expression in MCF cells. Our study demonstrates that exposure to PM_2.5_ induces crosstalk between macrophages and cardiac fibroblasts, leading to fibrosis activation. Furthermore, ERS and the TXNIP-NLRP3 pathway play crucial roles in this process.

We are also attempting to further investigate the role of PM_2.5_ in inducing cardiomyocyte death through the ERS/TXNIP/NLRP3 signaling pathway. Cardiomyocyte death can trigger an inflammatory response, leading to the activation of fibroblasts, which replace the dead cardiomyocyte with fibrous tissue, thereby contributing to cardiac fibrosis [56]. There have been studies on the effects of PM_2.5_ on cardiomyocyte death, but the role of the ERS/TXNIP/NLRP3 pathway in this process remains unclear [39]. In this study, the ROS levels in H9c2 cells of the PM_2.5_ group were elevated, while the expression of Sod1 and Nrf-2 proteins was reduced. Furthermore, the expression of inflammatory factors NF-κB, IL-6, and IL-1β was increased. The results demonstrate that PM_2.5_ exposure leads to elevated levels of ROS in H9c2 cells, inducing oxidative stress and inflammatory responses. The detection results of the ERS/TXNIP/NLRP3 signaling pathway in H9c2 cells indicate that it plays a role in the PM_2.5_-induced death of H9c2 cells. The application of the ERS inhibitor 4-PBA and the TXNIP inhibitor SRI-37330 alleviated the elevated ROS levels, oxidative stress, inflammatory response, and apoptosis induced by the PM_2.5_ exposure in H9c2 cells, as well as mitigated the ERS/TXNIP/NLRP3 signaling pathway-related apoptosis. The above results indicate that ERS and TXNIP induced by PM_2.5_ result in H9c2 cell death and then aggravate cardiac fibrosis.

**Figure 9:**
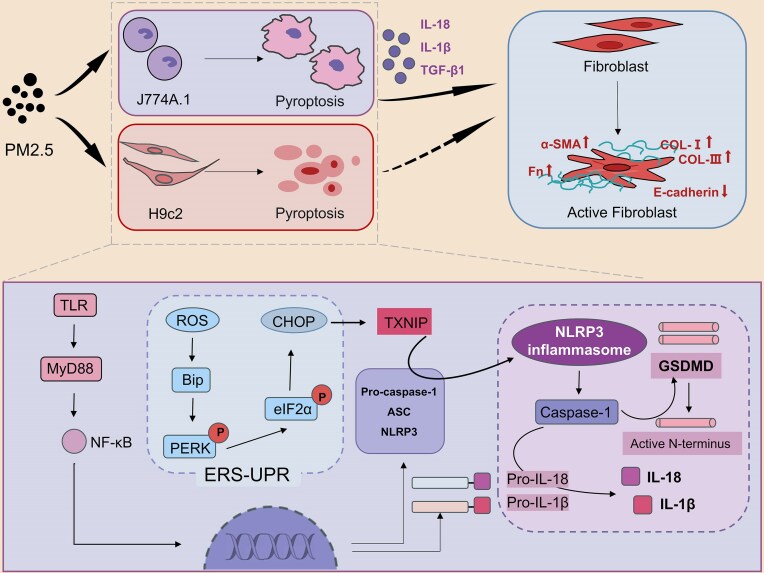
The schematic illustration of PM_2.5_ induces cardiac fibrosis via ERS/TXNIP/NLRP3 pathway in cells crosstalk. PM_2.5_ exposure induces ERS in macrophage cell line J774A.1 and cardiomyocyte cell line H9c2, and promotes pyroptosis through the activation of TXNIP. Pyroptosis triggers the release of cytokines such as IL-18, IL-1β, and TGF-β1 by macrophages. The release of these cytokines can activate cardiac fibroblasts, leading to the upregulation of fibrosis markers such as α-SMA, COL-I, and COL-III, which indicates the differentiation of fibroblasts into myofibroblasts, thereby promoting the progression of cardiac fibrosis. PM_2.5_ promotes the transcriptional upregulation of Pro-Caspase-1, ASC, and NLRP3 in the nucleus through the TLR/MyD88/NF-κB pathway, as well as the expression of Pro-IL-18 and Pro-IL-1β. On the other hand, after exposure to PM_2.5_, the stimulation of cells induces a high-level change in ROS. Excessive ROS triggers the occurrence of ERS, leading to the dissociation of Bip from the endoplasmic reticulum, which in turn activates the phosphorylation of PERK. This activation results in the phosphorylation of eIF2α, subsequently causing an upregulation of CHOP expression. The expression of TXNIP, regulated by CHOP, is upregulated, promoting the assembly of the NLRP3 inflammasome consisting of Pro-Caspase-1, ASC, and NLRP3, and facilitating the activation of Pro-Caspase-1 to Caspase-1. On one hand, Caspase-1 activates Pro-IL-18 and Pro-IL-1β, converting them into their active forms, IL-18 and IL-1β. On the other hand, Caspase-1 induces the N-terminal cleavage of GSDMD, thereby promoting the formation of membrane pores. Consequently, the activated cytokines, including IL-18 and IL-1β, are released from the cell.

This study establishes PM_2.5_ exposure models in macrophages, cardiomyocytes, and cardiac fibroblasts. Through extensive experimentation, it confirms that the ERS/TXNIP/NLRP3 signaling pathway plays a crucial role in the development of cardiac fibrosis in these different cell types and in cell-to-cell action (Fig. 9). Notably, our previous *in vivo* study demonstrated that PM₂.₅ exposure induces cardiac fibrosis in animal models via the ERS/TXNIP/NLRP3 signaling pathway, thereby supporting the mechanisms identified in the present *in vitro* study and providing a foundation for further investigation [57]. Our experimental results suggest that PM₂.₅-induced cardiac fibrosis involves coordinated interactions among multiple cell types. PM₂.₅ exposure promotes cardiomyocyte death through activation of the ERS/TXNIP/NLRP3 signaling pathway. Previous studies have shown that dying cardiomyocytes release damage-associated molecular patterns (DAMPs), which subsequently activate macrophages and cardiac fibroblasts [58]. As a central component of cell-to-cell action, the action between macrophages and cardiac fibroblasts plays a crucial role in the process of fibrosis induced by PM_2.5_. Although epigenetic modifications were not directly assessed in the present study, accumulating evidence suggests that PM_2.5_-induced oxidative stress can trigger epigenetic alterations, including DNA methylation and histone acetylation [59]. These changes may contribute to the sustained activation of genes associated with the ERS/TXNIP/NLRP3 pathway, thereby amplifying intercellular signaling [60]. Further elucidation of epigenetic regulation in this context warrants future investigation.

ERS and TXNIP are central to the molecular mechanism crosstalk, as they play key roles not only in the ERS/TXNIP/NLRP3 signaling pathway but also in influencing other related mechanisms. The results of this study indicate that following the inhibition of ERS expression, high levels of ROS, oxidative stress, and inflammatory responses were attenuated to varying extents. This suggests a correlation between ERS and the generation of ROS, oxidative stress, and inflammatory responses, which is consistent with the findings of related studies [61]. The ERS-related protein CHOP not only enhances the expression of TXNIP by regulating the expression of downstream genes, but both proteins also jointly participate in the regulation of cellular oxidative stress, pyroptosis, and apoptosis. Specifically, the administration of 4-PBA leads to a downregulation of CHOP expression, accompanied by a significant reduction in TXNIP expression, thereby alleviating the extent of pyroptosis and apoptosis (Figs 7 and 8). The application of the TXNIP inhibitor SRI-37330 further alleviated the extent of oxidative stress and apoptosis. This may be associated with the formation of the TXNIP/Trx2 complex under conditions of ROS overload. The TXNIP/Trx2 complex can induce the phosphorylation of apoptosis signal-regulating kinase 1 (ASK1). Phosphorylated ASK1 stimulates the release of cytochrome c (Cyto c), which in turn triggers the expression of Caspase-3, ultimately leading to apoptosis [23]. The results of this study indicate that the expression of Caspase-3 significantly decreased after treatment with SRI-37330, possibly through this pathway. Notably, accumulating evidence indicates that NLRP3 inflammasome expression is subject to epigenetic regulation, particularly through alterations in DNA methylation patterns. In addition, a study of diabetic cardiomyopathy demonstrated that METTL14 attenuates cardiomyocyte pyroptosis and downregulates NLRP3 expression by modulating m6A modification of the long non-coding RNA TINCR [62]. This question will be addressed in future studies using organoid models, gene knockout, RNA interference, and epigenetic pharmacological approaches in combination with animal experiments, to further determine whether epigenetic modifications serve as regulatory mechanisms of the ERS/TXNIP/NLRP3 signaling pathway in the initiation and progression of PM_2.5_-induced cardiac fibrosis.

## Materials and methods

### PM_2.5_ preparation

PM_2.5_ was collected on a glass fiber filter (Whatman, Little Chalfont, Buckinghamshire, UK) using a high-volume sampler (Waltham, MA, USA) from October 2022 to April 2023 in Weifang (China). After preparation and extraction [37], the sample was stored in a −20°C freezer. A PM_2.5_ stock solution with a concentration of 4 mg/ml was prepared using a basal cell culture medium (DMEM + 1% P/S). Following 30 min of UV irradiation, the stock solution was stored at −20°C. Before use, it was thawed and thoroughly mixed by ultrasonication for experimental purposes.

### Cell lines and culture

**Cell culture**. Mouse bone marrow mononuclear macrophage (J774A.1) and rat cardiomyocytes (H9c2) were purchased from Wuhan Pricella Biotechnology Co., Ltd. And we got the immortalized mouse myocardial fibroblasts (MCF) from Wuxi Newgain Biotechnology Co., Ltd. Cells were maintained in DMEM in the presence of 10% FBS and 1% P/S, and were cultured in an incubator in 5% CO_2_ at 37°C. Cells were subcultured or plated every 2 days upon reaching ~80% confluence.

**PM_2.5_ exposure**. The intermediate PM_2.5_ concentration was defined as that causing ~50% loss of cell viability. Higher and lower doses were set as multiples of this concentration, with final selection further informed by ROS and LDH measurements. J774A.1 were treated with different dosage: the PM_2.5_ low dose (50 μg/ml), the PM_2.5_ medium dose (100 μg/ml), and the PM_2.5_ high dose (200 μg/ml). The exposure doses of PM_2.5_ for each H9c2 groups were: low exposure dose (25 μg/ml), medium exposure dose (50 μg/ml), and high exposure dose (100 μg/ml). Both J774A.1 and H9c2 cells mentioned above were exposed to PM2.5 for 24 h.

**J774A.1 stimulation**. To induce the activation of inflammatory pyroptosis in J774A.1, we treated cells by the LPS (100 ng/ml) and the adenosine 5′-triphosphate (ATP) (3 mM) for 24 h. After the positive control treatment (LPS + ATP), we verify the J774A.1 whether or not occurs inflammatory pyroptosis through observation of the cell morphology, western blotting, and ELISA assay.

**Indirect co-processing of macrophages-myocardiofibroblasts**. We collected the culture supernatant of J774A.1 after PM_2.5_ exposure and positive control treatment. To ensure that cells and cellular debris were cleaned up, we centrifugated at low speed (700 rpm) for 10 min. Next, we used the ELISA assay to test the content of the cytokines in the collected culture supernatant, which for the indirect co-processing model of macrophages-myocardial fibroblasts. Besides, after the cell morphology observation and MTT assay, the time of treatment was decided to be 24 h.

**ERS and TXNIP inhibition**. MTT tests were used to determine the ERS and TXNIP inhibitor dosages, according to the concept of utilizing the maximum concentration that did not impair cell viability. J774A.1 cells were treated with 2 mM 4-PBA and 2 μM SRI-37330. H9c2 cells were given 2 mM 4-PBA and 1 μM SRI-37330, respectively. Both J774A.1 and H9c2 cells mentioned above were exposed to PM2.5 for 24 h.

### MTT assay

The cells were seeded in 96-well plates at 5 × 10^4^/ml. We added the regents, including different dosages of PM_2.5_, LPS, ATP, 4-PBA, SRI-37330, and cell culture supernatant of J774A.1. After the treatment which was planned, the MTT (Solarbio, M8180) detection solution (0.5 mg/ml) that diluted with culture medium incubate cells 200 μl every plate. After incubating for 4 h at 37°C in an incubator protected from light, we discarded the supernatant and added 150 μl DMSO, and then shook the plates on a shaker for 10 min to completely dissolve the crystals. The cell activity was acquired via using Multiskan FC microplate reader (Thermo Fisher Scientific, Waltham, MA, USA) at an absorption wavelength of 570 nm.

### ROS staining

We cultivated J774A.1 and H9c2 in six-well plates and cultured overnight. After the treatment of different groups, the culture solution was removed. The 10 μM DCFH-DA (Beyotime Biotechnology, S0035M) was added for 1 ml each well. After incubating for 20 min at 37°C in an incubator protected from light, we washed three times with culture medium and observed using a fluorescent inverted microscope (Leica; EX/EM: 488/525).

### JC-1 staining

The detection of changes in mitochondrial membrane was accomplished with the mitochondrial membrane potential assay kit with JC-1 (Beyotime Biotechnology, C2006). The cells were seeded in a confocal dish and cultured overnight. After the treatment of each group, we added 1 ml JC-1 detection solution each dish to complete the fluorescent staining, and incubated for 20 min at 37°C in an incubator protected from light. After washing with the JC-1 staining buffer, we added 2 ml cell culture fluid and observed by a laser confocal microscope (Leica, Wetzlar, Ger­ many; Red, EX/EM: 585/590; Green, EX/EM: 514/529).

### Calcein/PI assay

The J774A.1 was cultivated in six-well plates and cultured overnight. After the PM_2.5_ exposure and the treatment of LPS + ATP, the culture solution was removed, and the plate was washed with PBS once. We added 100 μl Calcein/PI detection solution (Beyotime Biotechnology, C2015) in each well. After incubating for 30 min at 37°C in an incubator protected from light, we observed by a fluorescent inverted microscope (Leica; Red, EX/EM: 535/617; Green, EX/EM: 494/517).

### Annexin V-FITC assay

We detected the apoptosis of cells by using Annexin V-FITC Apoptosis Detection Kit (Beyotime Biotechnology, C1062). The J774A.1 and H9c2 were seeded in the six-well plate and cultured overnight. The culture solution was removed after the treatment of groups. According to the operating instructions, we added the Annexin V-FITC mixture, Annexin V-FITC, and the propidium iodide (PI) staining solution proportionally. Next, we incubated the plates at room temperature away from light for 20 min. Immediately afterwards, we observed by a fluorescent inverted microscope. The Annexin V-FITC is green fluorescent and PI is red fluorescent (Leica; Red, EX/EM: 535/617; Green, EX/EM: 494/517).

### IF staining

We seeded the round coverslip of MCF in the six-well plates and cultured overnight. After the treatment of the supernatant of J774A.1 for 24 h, the plates were washed with PBS three times. To fix the cells, we used the 4% paraformaldehyde (Biosharp, BL539A) to cover the round coverslip for 30 min at room temperature. Subsequently, the paraformaldehyde was discarded, and the plates were washed with PBS three times. The 0.2% Triton X-100 (Beyotime Biotechnology, P0096) was formulated by PBS, and added appropriate volume to the round coverslip for 15 min at room temperature. After that, we added the QuickBlock™ Blocking Buffer for Immunol Staining (Beyotime Biotechnology, P0260) for 25 min at room temperature. After removing the blocking buffer, the round coverslip was covered with ~50 μl primary antibodies, and were putted in wet box overnight at 4°C. We recovered the primary antibodies and washed with PBS three times the next day. After that, we added the Cy3-labeled Goat Anti-Rabbit IgG (H + L) (Beyotime Biotechnology, A0516) and Cy3-labeled Goat Anti-Mouse IgG (H + L) (Beyotime Biotechnology, A0521) ~20 μl for 1 h at room temperature. After washing the round coverslip with PBS three times, we placed the round coverslip on a slide that dropped of Antifade Mounting Medium with DAPI (Beyotime Biotechnology, P0131). We observed using a fluorescent inverted microscope.

### ELISA

The supernate was collected from the J774A.1 that after the different treatment, and was centrifuged at low speed (4000 rpm) for 20 min. After that, to detected the level of IL-18, IL-1β, and TGF-β1, we used the ELISA assay kit (Ruixin Biotech, RX203064M, RX203063M, and RX202402M) according to the instructions.

### Real-time quantitative PCR

The total RNA was extracted from the pellets of MCF cells treated with J774A.1 cell supernatant by Trizol (solarbio, R1100). We acquired the cDNA by using the First Strand cDNA Synthesis Kit with gDNA EZeraser (Beyotime Biotechnology, D7180L). The PCR reaction system was composed of SYBR Green qPCR Mix (2×) (Beyotime Biotechnology, D7260), Forward and Reverse Primer Mix, Template DNA, and RNase-Free Water. After fully mixing the reaction system, we detected the mRNA levels of samples using the T100 ThermalCycler PCR instrument (Bole, USA). The relative changes in mRNA levels were normalized to the relative changes in β-actin using the 2 ^−△△Ct^ method. Primer sequences used in this study are available in Supplementary Table 1.

### Western blotting assay

The culture medium in the six-well plate after treatment was removed, and the wells were washed three times with PBS. Subsequently, 200 μl of RIPA buffer (containing PMSF) was added, and the mixture was incubated on ice with gentle shaking for 30 min. The cells on the plate were carefully scraped off, and the cell suspension was transferred into a 1.5 ml centrifuge tube. The tubes were then placed in a low-speed centrifuge (1000 rpm, 10 min). After centrifugation, the supernatant was carefully aspirated and transferred into a new centrifuge tube. The protein concentration in the supernatant of cells from each group was determined using a BCA assay kit. Based on the results, the protein content of each group was normalized. An appropriate volume of RIPA buffer and 5X loading buffer was then added, followed by thorough mixing and centrifugation. The samples were subsequently boiled at 100°C for 5 min. An appropriate amount of cell protein sample and markers were loaded into the wells of an 8% or 12% SDS-PAGE gel. After electrophoresis, the gel was cut and placed onto a nitrocellulose (NC) membrane for transfer. Following the membrane transfer, the NC membrane was blocked and incubated overnight with the primary antibody (the information of antibodies in Supplementary Table 2) at 4°C. The next day, after washing with TBST, the membrane was incubated at room temperature with the secondary antibody (the information of antibodies in Supplementary Table 2). The results were then visualized using a chemiluminescent substrate and analyzed with an imaging system.

### Statistical analysis

All data reported are from at least three replicates per experimental group and presented as mean ± SD. Between-group comparisons were analyzed using *t*-tests or ANOVA, with least significant difference (LSD) *post hoc* tests for equal variances and Dunnett’s tests for unequal variances. A *P*-value < .05 was considered statistically significant (ns ≥ .05; *P < .05). Statistical analyses were performed using SPSS (version 26.0), while graphical visualizations were generated through GraphPad Prism (version 8.0).

## Supplementary Material

dvag015_Supplemental_File

## Data Availability

The datasets used and/or analyzed during the present study appear in the submitted article.
