# Supplementary material for: Mechanistic insights into PM2.5-induced cardiac fibrosis involving ERS/TXNIP/NLRP3-driven pyroptosis in multiple cell types
Source: Environ Epigenet. 2026 May 7;12(1):dvag015. doi: 10.1093/eep/dvag015 (PMC13370249; doi:10.1093/eep/dvag015)
Supplement: dvag015_Supplemental_File [file dvag015_supplemental_file.doc]

Supplementary Information for

**Mechanistic Insights into PM_2.5_-Induced Cardiac Fibrosis Involving ERS/TXNIP/NLRP3-Driven Pyroptosis in Multiple Cell Types**

Siqi Li^1#^, Xiaohong Li^1,2,3#^, Xiaolin Han^1,2,3#^, Mengxiao Luan^1,2,3^, Fengjiao Tan^1,2,3^, Yumei Liu^1,2,3^, Ruixi Zhou^1^, Wenbo Wu^1^, Chen Liu^1^, Limin Zhang^1^, Qin Wang^4^, Yingjie Zou^5^, Jinfeng Tan^5^, Li Yu^6,7^, Wanwei Li^1,2,3*^

^1^ School of Public Health, Shandong Second Medical University, Weifang, China, 261053

^2^ "Healthy Shandong" Major Social Risk Prediction and Governance Collaborative Innovation Center, Weifang, China, 261053

^3^ Key Laboratory of Health Inspection and Quarantine, Weifang, China, 261053

^4^ National Institute of Environmental Health of China CDC, Beijing, China, 100021

^5^ Weifang Ecological Environmental Monitoring Station, Weifang, China, 261044

^6^ School of Basic Medicine, Neurologic Disorders and Regeneration Repair Lab of Shandong Higher Education, Shandong Second Medical University, Weifang, China, 261053,

^7^ Basic Medical College, Shandong Medical And Pharmaceutical University, Yantai, China，264003

^#^ These authors contribute equally to this study

^*^Correspondence:

Wanwei Li：[lilili127@163.com](mailto:lilili127@163.com)


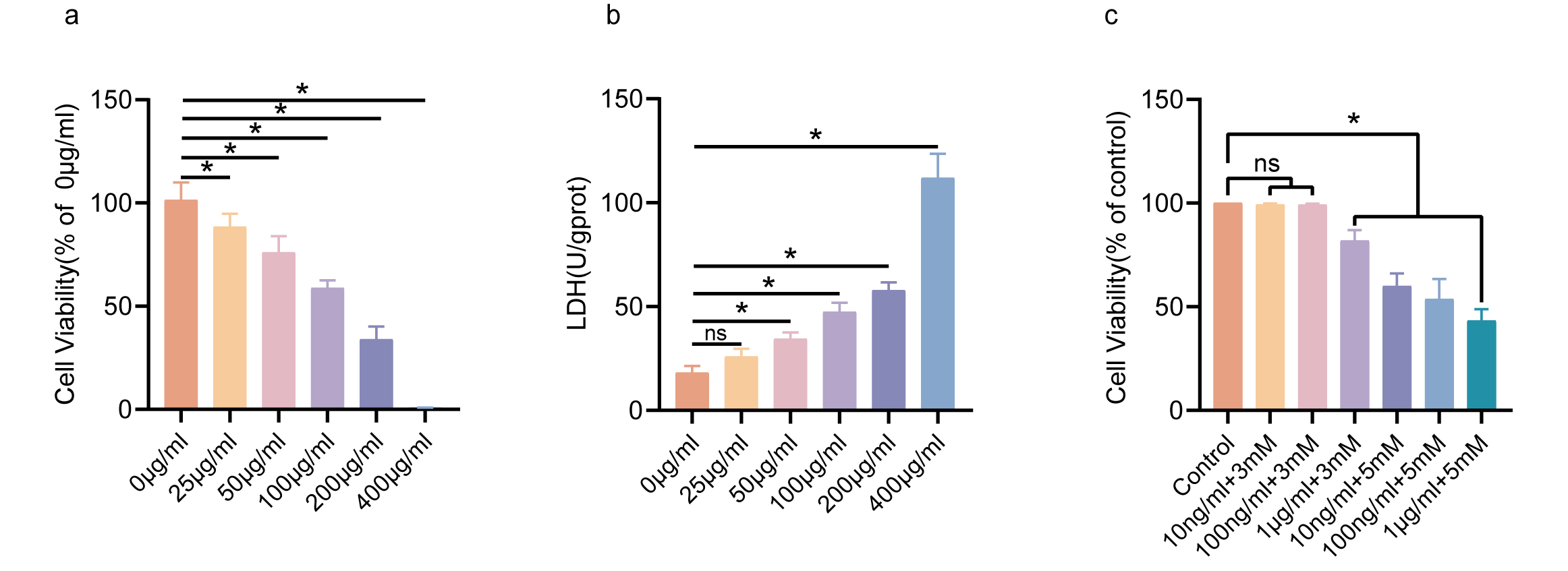


**Supplementary Fig. 1** **The impairment of different dosages of PM2.5 and active control treatment on the J774A.1.** (a)The cell viability of J774A.1 cells and (b) The LDH release levels of J774A.1 cells after different dosages of PM2.5. (c) The cell viability of J774A.1 cells after different dosages of active control treatment (LPS+ATP). The data shown as mean±SD (n = 3). ns，*P* ≥0.05; **P* ＜0.05.


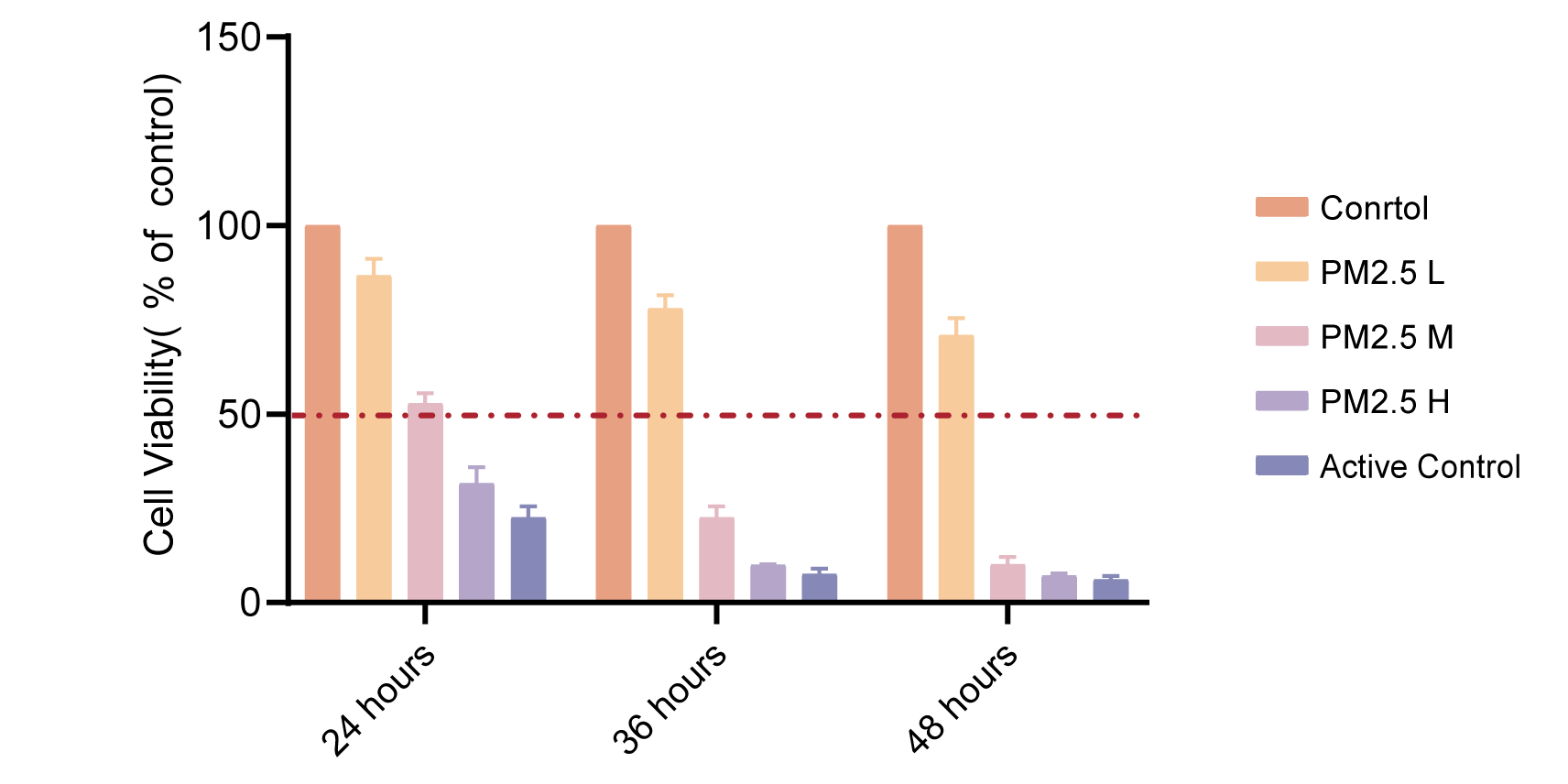


**Supplementary Fig. 2 The cell viability of MCF after different time of J774A.1 cells culture supernatant treatment.** The data shown as mean±SD (n = 3).


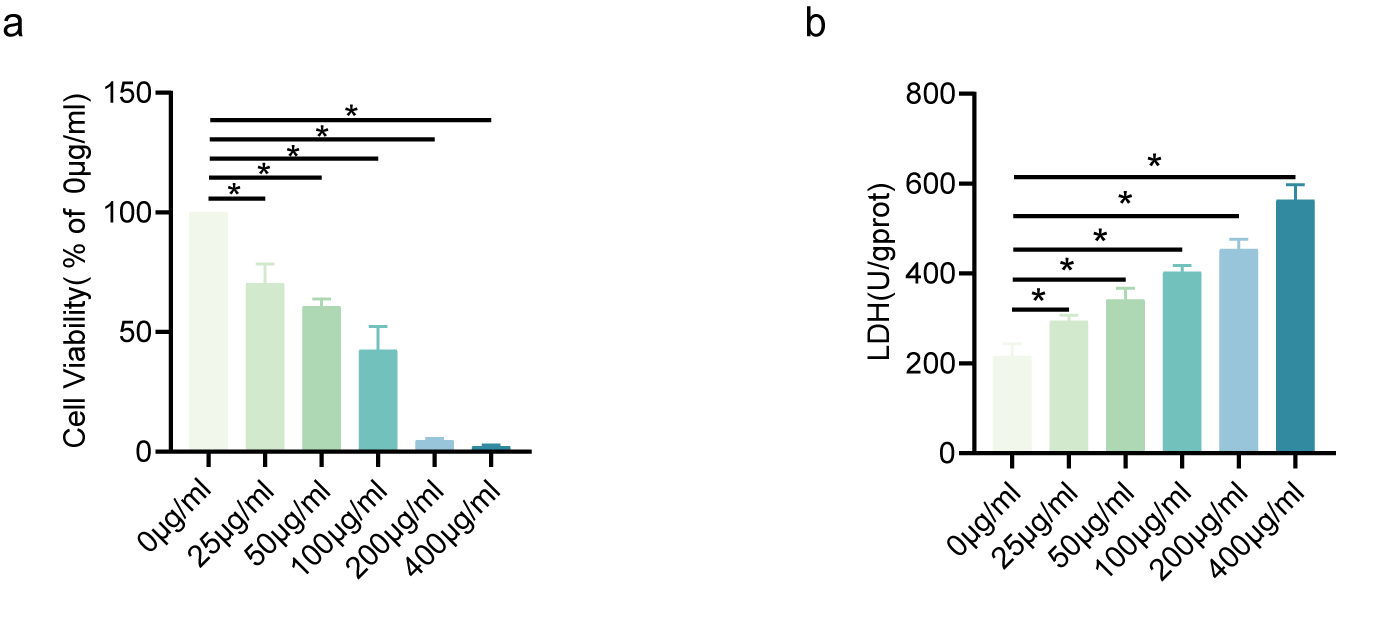


**Supplementary Fig. 3 The impairment of different dosages of PM2.5 on H9c2 cells.** (a)The cell viability and (b) The LDH release levels of H9c2 cells after different dosages of PM2.5. The data shown as mean±SD (n = 3). **P* ＜0.05.


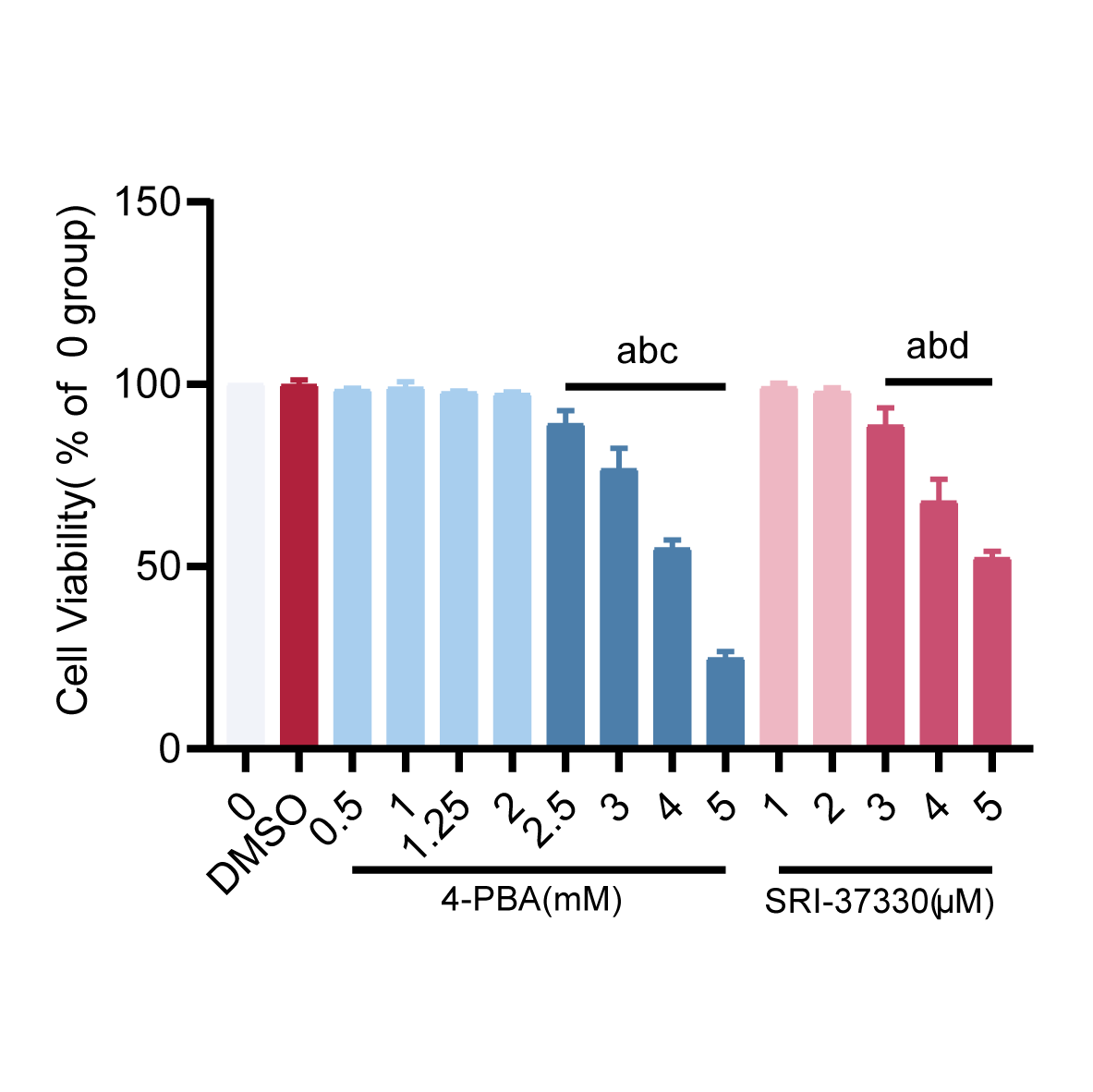


**Supplementary Fig. 4 The impairment of different dosages of 4-PBA and SRI-37330 on the cell viability of J774A.1 cells.** The data shown as mean±SD (n = 3). a, vs 0 group, *P* ＜0.05; b, vs DMSO group, *P* ＜0.05; c, vs 2mM 4-PBA group, *P* ＜0.05; d, vs 2μM SRI-37330 group, *P* ＜0.05.


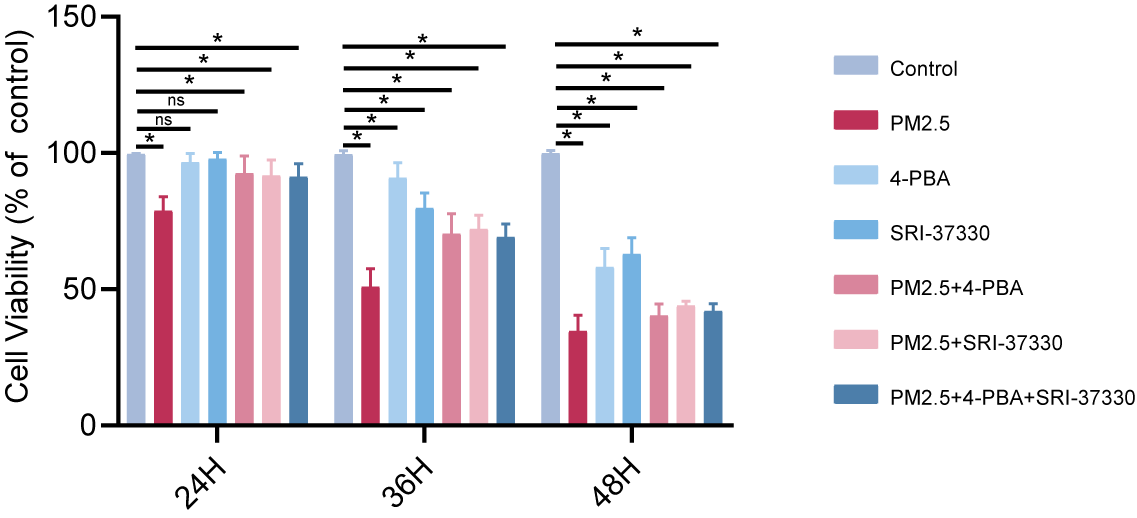


**Supplementary Fig. 5 The cell viability of MCF after different time of J774A.1 cells culture supernatant treatment (PM2.5, 4-PBA, and SRI-37330).** The data shown as mean±SD (n = 3). ns，*P* ≥0.05; **P* ＜0.05.

**
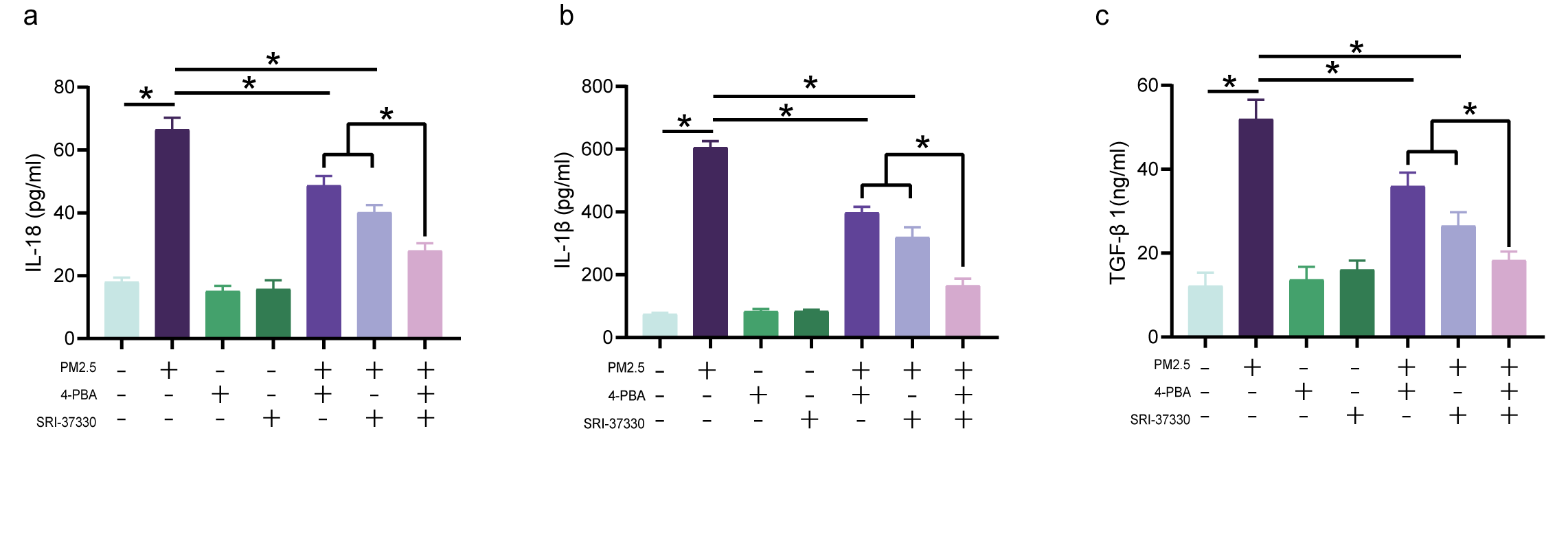
**

**Supplementary Fig. 6 Secretion of IL-18, IL-1β, and TGF-β1 by J774A.1 cells after PM2.5 activation ERS and TXNIP.** After the treatment of PM2.5, 4-PBA, and SRI-37330, the （a）IL-18, (b)IL-1β, and (c)TGF-β1 in J774A.1 cells supernatant was measured by ELISA. The data shown as mean±SD (n = 3). **P* ＜0.05.


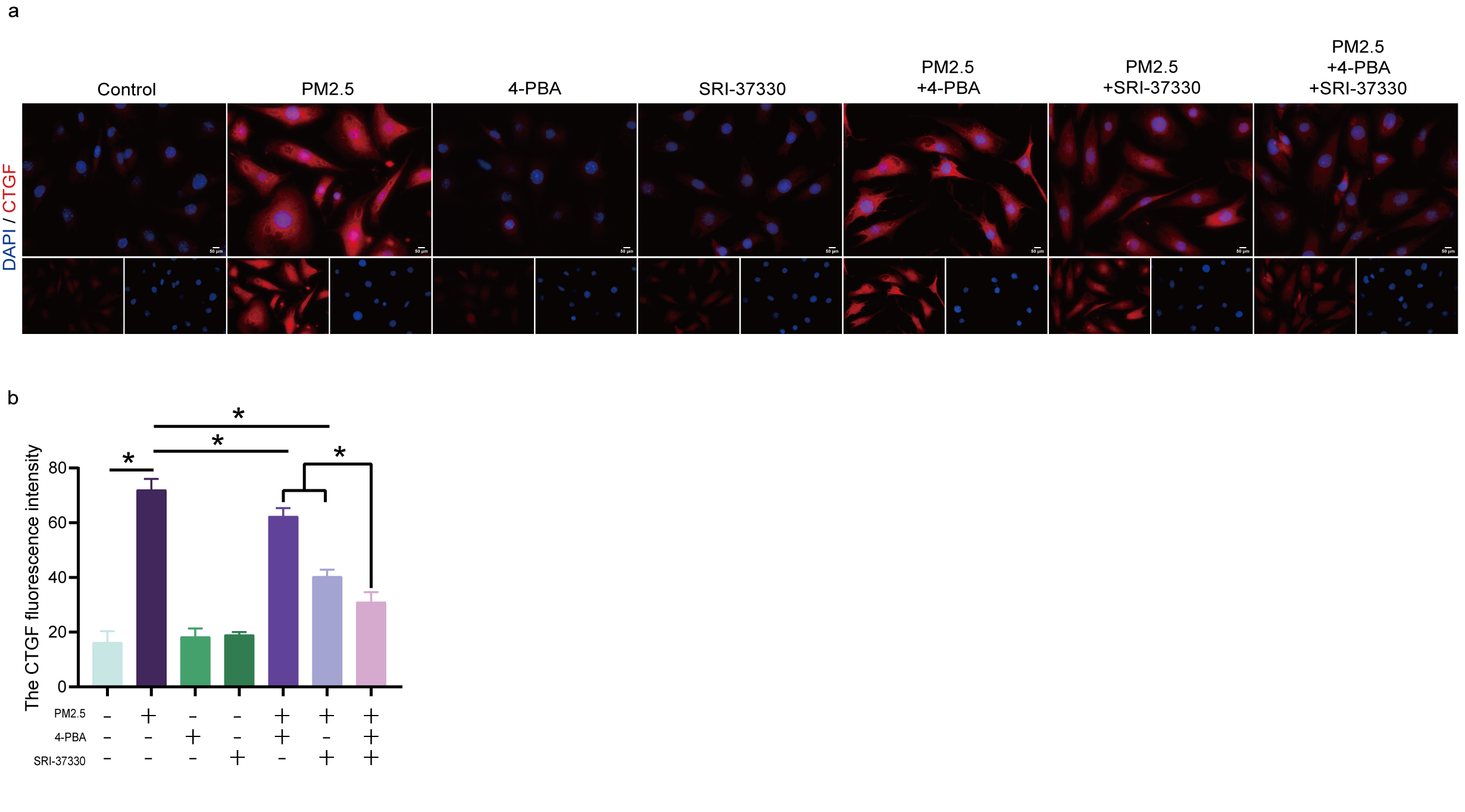


**Supplementary Fig. 7 The immunofluorescence analysis of CTGF in MCF after the treatment of J774A.1 cells culture supernatant.** The CTGF (a)microscopic results and (b)statistical analysis results. The data shown as mean±SD (n = 3). **P* ＜ 0.05.


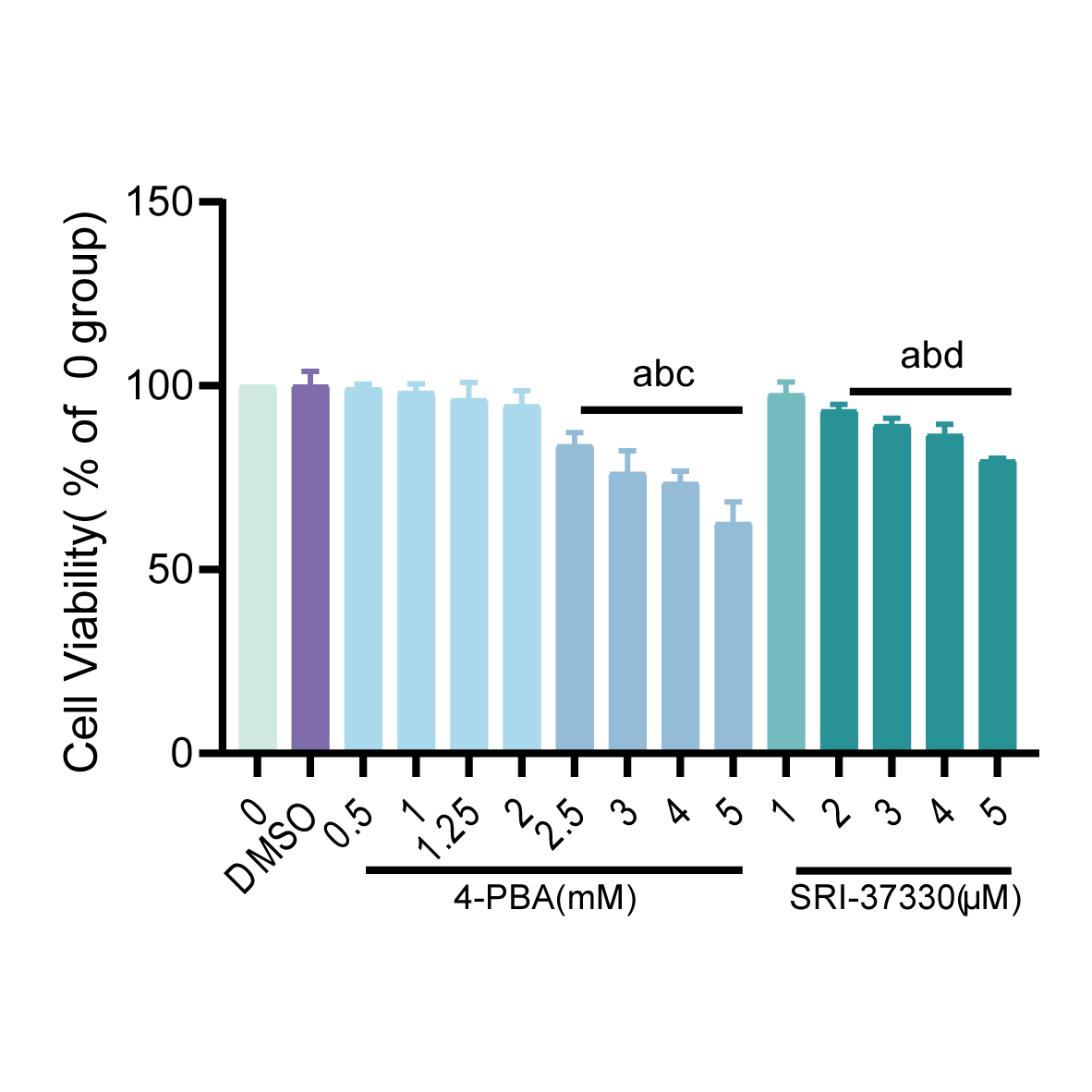


**Supplementary Fig. 8 The impairment of different dosages of 4-PBA and SRI-37330 on the cell viability of H9c2 cells.** The data shown as mean±SD (n = 3). a, vs 0 group, *P* ≤ 0.05; b, vs DMSO group, *P* ≤ 0.05; c, vs 2mM 4-PBA group, *P* ≤ 0.05; d, vs 1μM SRI-37330 group, *P* ≤ 0.05.

**Supplementary Table 1** Primer Sequences

| Target Gene | Forward Primer (5’to 3’) | Reverse Primer (5’to3’) |
| --- | --- | --- |
| TGF-β1 | 5’ - CTCCCGTGGCTTCTAGTG - 3’ | 5’- GCCTTAGTTTGGACAGGATCTG - 3’ |
| FN | 5’ - ATGTGGACCCCTCCTGATAGT - 3’ | 5’ - GCCCAGTGATTTCAGCAAAGG - 3’ |
| COL-Ⅰ | 5’ - GCTCCTCTTAGGGGCCACT - 3’ | 5’ - CCACGTCTCACCATTGGGG - 3’ |
| COL-Ⅲ | 5’ - CTGTAACATGGAAACTGGGGAAA - 3’ | 5’ - CTGTAACATGGAAACTGGGGAAA - 3’ |
| α-SMA | 5’ - GTCCCAGACATCAGGGAGTAA - 3’ | 5’ - TCGGATACTTCAGCGTCAGGA - 3’ |
| E-cadherin | 5’ - CAGGTCTCCTCATGGCTTTGC - 3’ | 5’ - CTTCCGAAAAGAAGGCTGTCC - 3’ |
| β-actin | 5’ - CATTGCTGACAGGATGCAGAAGG - 3’ | 5’ - TGCTGGAAGGTGGACAGTGAGG - 3’ |

**Supplementary Table 2** Antibodies Information

| Antibodies | Commercial sources | Dilutions |
| --- | --- | --- |
| Sod1 | Beyotime#AF8028 | 1:1000 |
| Nrf-2 | Beyotime#AF7623 | 1:1000 |
| NF-κB | Beyotime#AF0246 | 1:1500 |
| IL-6 | Abmart#TD6087 | 1:1000 |
| IL-1β | Abmart#P50520-1R1 | 1:1000 |
| PERK | Wanlei#WL03378 | 1:1500 |
| p-PERK | Beyotime#AF5902 | 1:1000 |
| CHOP | Wanlei#WL0080 | 1:1500 |
| Bip | Beyotime#AB310 | 1:1000 |
| NLRP3 | Beyotime#AF2155 | 1:750 |
| Caspase-1 | Beyotime#AF1681 | 1:1000 |
| TXNIP | Wanlei#WL05902 | 1:1500 |
| GSDMD-N | ABclonal#A20197 | 1:1000 |
| CRT | Beyotime#AF1666 | 1:2000 |
| Caspase-3 | Beyotime#AF5132 | 1:1000 |
| Caspase-9 | Beyotime#AC062 | 1:1000 |
| COL-Ⅲ | Affinity#AF5457 | 1:1000 |
| COL-Ⅰ | Affinity#AF1032 | 1:750 |
| E-cadherin | Beyotime#AF0138 | 1:750 |
| α-SMA | Affinity#AF7001 | 1:750 |
| TGF-β1 | Beyotime#AF0297 | 1:750 |
| CTGF | Beyotime#AF6582 | 1:1000 |
| β-actin | ABclonal#AC038 | 1:10000 |
| Anti-Rabbit IgG | Proteintech#SA00001-2 | 1:1000 |
| Anti-Rabbit IgG | Proteintech# SA00001-1 | 1:1000 |
